# *Lactobacillus plantarum* PFM 105 Promotes Intestinal Development Through Modulation of Gut Microbiota in Weaning Piglets

**DOI:** 10.3389/fmicb.2019.00090

**Published:** 2019-02-05

**Authors:** Tianwei Wang, Kunling Teng, Yayong Liu, Weixiong Shi, Jie Zhang, Enqiu Dong, Xin Zhang, Yong Tao, Jin Zhong

**Affiliations:** ^1^State Key Laboratory of Microbial Resources, Institute of Microbiology, Chinese Academy of Sciences, Beijing, China; ^2^School of Life Science, University of Chinese Academy of Sciences, Beijing, China; ^3^LongDa Foodstuff Group Co., Ltd, Laiyang, China

**Keywords:** *Lactobacillus plantarum*, antibiotics, weaning piglets, intestinal development, microbiota

## Abstract

*Lactobacillus plantarum* is a widespread bacterial species and is commonly used as a probiotic. *L. plantarum* PFM105 was isolated from the rectum of a healthy sow. Here we found that *L. plantarum* PFM105 showed probiotic effect on weaning piglets in which intestinal inflammation and unbalanced gut microbiota happened frequently. *L. plantarum* PFM105 was identified to improve the growth of weaning piglet and promote the development of small intestinal villi. Antibiotics are often used in weaning piglet to prevent intestinal infection and promote the growth of animal. We found that weaning piglets feeding with *L. plantarum* PFM105 showed similar growth promotion but decreased diarrhea incidence compared with those feeding with antibiotics. High-throughput sequencing was used to analyze the gut microbiota in weaning piglets treated with *L. plantarum* PFM105 or antibiotics. The relative abundance of beneficial microbes Prevotellaceae and Bifidobacteriaceae were increased in colon of weaning piglet feeding *L. plantarum* PFM105, while antibiotics increased the relative abundance of bacteria associated with pathogenicity, such as *Spirochaeta* and *Campylobacteraceae*. *L. plantarum* PFM 105 increased indicators of intestinal health including serum levels of IgM, IL-10, and TGF-β, and colonic levels of SCFAs. We found strong correlations between the alterations in gut microbiota composition caused by feeding antibiotics and probiotics and the measured growth and health parameters in weaning piglets. The addition of *L. plantarum* PFM105 could significantly increase the relative abundance of metabolic genes which may important to intestinal microbiota maturation. Altogether, we demonstrated here that *L. plantarum* PFM 105 could promote intestinal development through modulation of gut microbiota in weaning piglets.

## Introduction

Weaning is a critical and stressful event in the life cycle of mammals, including pigs, and is frequently associated with severe enteric infections and subsequent overuse of antibiotics (Gresse et al., 2017; Lariviere-Gauthier et al., 2018). Such periods of multiple stressors may induce transient anorexia, intestinal inflammation, and unbalanced gut microbiota (Su et al., 2008). The weaning transition generally causes gastrointestinal (GI) infections, mainly by opportunistic pathogens, such as Proteobacteria, which are associated with the death of around 17% of piglets born in Europe in each year (Lalles et al., 2007; Gresse et al., 2017). In China, about 24 million weaning piglets die from bacterially induced diarrhea annually, resulting in economic losses of 12 billion yuan each year (Yin, 2010).

Antibiotics have been recognized as one of the most successful therapies in both human and veterinary medicine, but they can lead to the development of resistant bacterial strains within human and animal gut microbiota (Toutain et al., 2016). In-feed antibiotics can also reduce α diversity and cause shifts in gut microbiota due to their wide spectrum activity and their potential ability to kill or prevent the growth of both pathogenic and beneficial microbes (Looft et al., 2012; Neuman et al., 2018). Prolonged use of subtherapeutic doses of antibiotics can increase opportunities for pathogenic microorganisms to colonize and trigger diseases (Schokker et al., 2014). Overzealous feeding of antibiotics to food animals can lead to the emergence of antibiotic-resistant microbes (Modi et al., 2014; Gao et al., 2017). Antibiotic apramycin sulfate is widely used to prevent piglet diarrhea due to its broad-spectrum antibacterial activity (Herrero-Fresno et al., 2016). However, apramycin administration is likely driving the increasing occurrence of apramycin/gentamicin cross-resistance of *Escherichia coli* (Jensen et al., 2006) and *Salmonella enterica* serotype *Typhimurium* (Lim et al., 2013) in swine. The use of apramycin may also lead to enhanced spread of gentamicin-resistant *E. coli* (Herrero-Fresno et al., 2016). These processes turn food animal systems into reservoirs of antibiotic resistance genes, which can transfer to the human population through consumption and lead to serious public health problems (Modi et al., 2014; Toutain et al., 2016). Abuse of human and animal antibiotics has also led to the development of antibiotic-associated diarrhea. As a result, many countries are banning or have banned the inclusion of antibiotics in swine diets as growth promoters (Samanidou and Evaggelopoulou, 2008; Thacker, 2013).

During past two decades, numerous studies have focused on the development of alternatives to antibiotics to maintain swine health and performance (Thacker, 2013; Wang J. et al., 2018). The most widely researched non-antibiotic alternatives include probiotics, prebiotics, acidifiers, and essential oils (Valeriano et al., 2016; Gresse et al., 2017; Wang W. et al., 2018). Among these alternatives, probiotics have higher potential to act as feed additives against pathogens (Gresse et al., 2017; Azad et al., 2018). Probiotics are defined as “a live microorganism that, when administered in adequate amounts, confers a health benefit on the host,” and are generally recognized as safe (GRAS) (FAO/WHO, 2002). *Lactobacillus plantarum* is a bacterium used as a probiotic, and is found in diverse ecological niches, such as mammal gastrointestinal tracts, dairy products, and vegetables. It has high adaptability and diversity of metabolic pathways (Seddik et al., 2017). *L. plantarum* has many probiotic characteristics including the ability to ferment a broad spectrum of plant carbohydrates, growth to high densities, tolerance of bile salts and low pH, and antagonistic potential against intestinal pathogens (Suo et al., 2012; van den Nieuwboer et al., 2016). *L. plantarum* ZJ316 can improve pig growth and pork quality, likely through inhibiting the growth of opportunistic pathogens and promoting increased villus height, rather than by altering the gut bacterial community (Suo et al., 2012). The metabolite combinations of mixed *L. plantarum* can improve growth performance and increase the population of gut lactic acid bacteria (LAB) and concentration of fecal short-chained fatty acids (SCFAs) of postweaning piglets (Thu et al., 2011). *L. plantarum* JC1 can increase villus height and the number of goblet cells, and improve the immune and inflammatory response by reducing intraepithelial lymphocytes and plasma TNF-α (Guerra-Ordaz et al., 2014). Although *L. plantarum* strains appear to have a high potential for replacement of antibiotics, few published studies have examined the effects of *L. plantarum* and antibiotics on weaning piglets.

In this study, we isolated the strain PFM105 from the rectum of a healthy sow and identified it as *L. plantarum* using 16S rDNA. We evaluated the effects of *L. plantarum* PFM105 and antibiotics on growth performance, clinical status, and intestinal morphology in weaning piglets. The colonic microbiota composition, metabolic capacity and the potential link between alterations in gut microbiota composition and health parameters in piglets feeding PFM 105 or antibiotics were also assessed. Weaning piglets feeding with *L. plantarum* PFM 105 showed elevated intestinal health and improved gut microbiota rather than those feeding with or without antibiotics.

## Results

### Growth Performance and Clinical Status

The weaning piglets were grouped as NC group (the negative control group) in which the piglets were fed the base diet without any antibiotics or probiotics, PC group (positive control group) in which the piglets were fed the base diet plus antibiotics, and LP group in which the piglets were fed the base diet plus probiotic *L. plantarum* PFM105. All the tested weaning piglets were fed for 21 days. During the first 2 weeks, there were no significant differences among the different groups in the metrics of average daily gain (ADG) and feed conversion ratio (FCR) (Table 1). However, during the third week, although there was no significant difference in the average daily feed intake (ADFI) in three groups, the ADG (*P* < 0.05) was significantly increased and the FCR was trend toward reduced in piglets in LP group compared to those in NC group (Table 1). These results demonstrated that *L. plantarum* PFM 105 might improve piglet growth performance while antibiotics did not.

**Table 1:** Effects of probiotics and antibiotics on weaning piglet performance, diarrhea incidence, and mortality.

Items	NC	PC	LP	*P* Value
Initial body weight (kg)	8.22 ± 0.35	8.26 ± 0.45	8.2 ± 0.46	0.9596
**Day 1–14**				
ADG (g/d)	97.6 ± 16.8	117 ± 19.5	101 ± 10.5	0.6689
ADFI (g/d)	208 ± 17.7	206 ± 15.5	215 ± 7.46	0.8693
FCR	2.28 ± 0.362	1.59 ± 0.23	2.22 ± 0.2	0.1726
**Day 14–21**				
ADG (g/d)	367 ± 17.4^b^	389 ± 22.6^ab^	440 ± 8.26^a^	0.0336
ADFI (g/d)	506 ± 25.9	512 ± 19.1	524 ± 60	0.8608
FCR	1.31 ± 0.06	1.29 ± 0.03	1.23 ± 0.06	0.1969
**Day 1–21**				
ADG (g/d)	187 ± 18.9	206 ± 11.6	214 ± 10.7	0.6943
ADFI (g/d)	308 ± 19.8	306 ± 15.7	318 ± 11.5	0.8283
FCR	1.58 ± 0.05	1.62 ± 0.09	1.53 ± 0.06	0.512
Fecal score	0.33 ± 0.04^a^b	0.45 ± 0.06^a^	0.28 ± 0.04^b^	0.0106
Diarrhea Incidence (%)	4.31 ± 0.98^a^b	5.26 ± 0.9^a^	3.12 ± 0.4^b^	0.031
Mortality (%)	4.17%	2.08%]	0.00%	0.36

*ADG, average daily gain; ADFI, average daily feed intake; FCR, feed conversion ratio. Different superscript letters within a row represent statistically significant differences among groups (*P* < 0.05). The fecal score of 0 corresponded to firm and dry, 1 to pasty, 2 to thick and fluid, and 3 to watery. The fecal score was calculated as the sum of the diarrhea score over the period divided by the number of piglet days in the period. The incidence of diarrhea (%) was calculated as the sum of the total number of diarrheal piglets over the period divided by the number of piglet days in the period multiplied by 100. The mortality (%) was calculated as the sum of the total number of dead piglets over the period divided by the number of piglets multiplied by 100.*

Piglets of the PC group did not show obvious difference in fecal score and diarrhea incidence with those of NC group. While piglets in LP group showed significantly decreased fecal score (*P* = 0.01) and diarrhea incidence (*P* = 0.01) compared to piglets in PC group (Table 1). Antibiotics (apramycin sulfate) and *L. plantarum* PFM105 reduced mortality of weaning piglets by 2.08 and 4.17%, respectively, compared to that of the NC group (Table 1). Thus, both *L. plantarum* PFM105 and antibiotics could reduce piglet mortality, but compared to antibiotics, *L. plantarum* PFM105 improved the clinical performance of piglets by reducing incidence of diarrhea and mortality.

### Effects of Probiotics and Antibiotics on Intestinal Morphology

Weaning stress is known to induce remarkable morphological alterations in the small intestine, such as villus atrophy and crypt hyperplasia (Montagne et al., 2007; Gresse et al., 2017). *L. plantarum* PFM105 treatment significantly increased the villus length over that of the NC (*P* = 0.0445) and PC groups (*P* = 0.0209), but had little effect on the crypt depth and the ratio of villus to crypt in the jejunum (Table 2). *L. plantarum* PFM105 treatment showed a trend toward increased villus length over the antibiotic treatment (*P* = 0.0518) in the ileum (Table 2), while it had little effect on crypt depth and the ratio of villus to crypt in the ileum. Antibiotics had no effect on villus length, crypt depth, or the ratio of villus to crypt in piglet small intestine. These results demonstrated that *L. plantarum* PFM 105 may promote the development of small intestinal villi, while antibiotics do not.

**Table 2:** Effects of probiotics and antibiotics on small intestine villus height (μm) and crypt depth (μm).

Position	Items	NC	PC	LP	*P* Value
**Jejunum**	Villus height	545 ± 32.9^b^	527 ± 39.2^b^	676 ± 33.8^a^	0.0157
	Crypt depth	202 ± 12.5	234 ± 22	237 ± 17	0.3211
	VH/CD	2.76 ± 0.21	2.39 ± 0.26	2.89 ± 0.11	0.1524
**Ileum**	Villus height	356 ± 24.5	325 ± 26.6	423 ± 29.4	0.0585
	Crypt depth	209 ± 10.9	199 ± 14.3	206 ± 10.7	0.8328
	VH/CD	1.71 ± 0.12	1.68 ± 0.17	2.09 ± 0.21	0.2874

*VH/CD, ratio of villus height to crypt depth. Different superscript letters within a row represent statistically significant differences among groups (*P* < 0.05).*

### Summary of Bacterial Community Richness and Biodiversity and β-Diversity

The microbiota of colonic contents in the three groups of piglets was analyzed by sequencing the bacterial 16S rDNA V3+V4 region. High-throughput pyrosequencing of the samples (*n* = 6) produced a total of 1,365,826 raw reads. After removing the low-quality sequences, 468,232 clean tags were identified as a total of 484 operational taxonomic units (OTUs) present in at least six samples. This sequencing depth almost reflected the total microbial species richness, and the majority of OTUs were present at low abundance, as demonstrated by the rarefaction, Shannon index, and rank abundance curves (Supplementary Figure S1). There were 385, 465, and 384 OTUs obtained from the NC, PC, and LP groups, respectively, of which 337 were common across the three experimental groups (Supplementary Figure S2). Moreover, a total of 71 unique OTUs were found within the NC, PC, and LP groups (5, 60, and 6, respectively). The Simpson alpha diversity index was higher for the LP group than for the NC group (*P* = 0.03). The Shannon, Chao 1, observed species (OS), and ACE values were not affected by treatment with either *L. plantarum* PFM105 or antibiotics (Table 3). To analyze the β-diversities of the colonic samples, we compared the Unweight Unifrac distances among colonic content samples collected from piglets. The microbial community structures of the NC and LP groups were mixed in the hierarchical clustering tree, while they were clearly distinguished from the PC group (Supplementary Figure S3A). Principal component analysis (PCA) based on phylum level (Supplementary Figure S3B) and OUT level (Supplementary Figure S3C) revealed that the gut microbiota in the NC and LP groups segregated from that of the PC group. The structure of the gut microbiota of piglets in the PC group was altered by use of antibiotics, while the probiotic *L. plantarum* PFM 105 did not detectably affect the gut microbiota structure.

**Table 3:** Alpha diversity indices of the colonic microbiota of weaning piglets.

Group	Coverage (%)	Richness estimator			Diversity index	
		Chao1	OS	ACE	Shannon	Simpson
**NC**	>99	255 ± 67.7	227.7 ± 59.7	253.7 ± 66.2	3.62 ± 0.15	0.052 ± 0.008^b^
**PC**	>99	326.7 ± 54.3	295 ± 55.7	321.2 ± 52.7	3.83 ± 0.48	0.059 ± 0.03^ab^
**LP**	>99	272.9 ± 49.2	245.5 ± 46.5	269.5 ± 47.1	3.41 ± 0.37	0.099 ± 0.039^a^

*Coverage percentage, richness estimators (Chao1, OS, and ACE), and diversity indices (Shannon and Simpson) were calculated using the mothur program. OS, observed species. Data in the same column that do not share a common superscript differ significantly (*P* < 0.05).*

### Characterization of the Colonic Microbiota of Piglets

**FIGURE 1:**
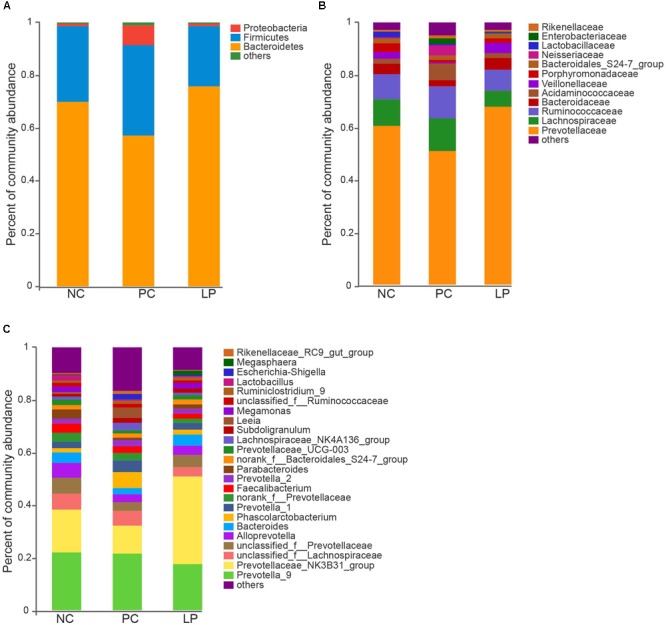
Effect of antibiotics and probiotic *Lactobacillus plantarum* PFM105 on gut microbiota composition in weaning piglets. Microbial community bar plot at the **(A)** phylum level, **(B)** family level, and **(C)** genus level.

We measured relative abundance of colonic microbiota that occurred as more than 1% of the microbiota at the phylum (Figure 1A), family (Figure 1B), and genus (Figure 1C) levels. The colonic microbiota was dominated by the phyla Bacteroidetes and Firmicutes (regardless of treatment), which constituted 66.7 and 27.4% of the total abundance, respectively (Figure 1A and Supplementary Table S1). Proteobacteria was also common, making up 3.6% of the total abundance (Supplementary Table S1). The dominant families within the phylum Bacteroidetes consisted of *Prevotellaceae, Bacteroidaceae, Bacteroidales* S24-7 group, *Porphyromonadaceae*, and *Rikenellaceae*. The main families within the phylum Firmicutes were *Lachnospiraceae, Ruminococcaceae, Acidaminococcaceae, Veillonellaceae*, and *Lactobacillaceae.* The dominant families belonging to phylum Proteobacteria were *Campylobacteraceae, Enterobacteriaceae*, and *Neisseriaceae* (Figure 1B). Other phyla (Tenericutes, Actinobacteria, Spirochaetae, Cyanobacteria, Fibrobacteres, Fusobacteria, and Deferribacteres) were present at very low relative abundances (Supplementary Table S1).

**FIGURE 2:**
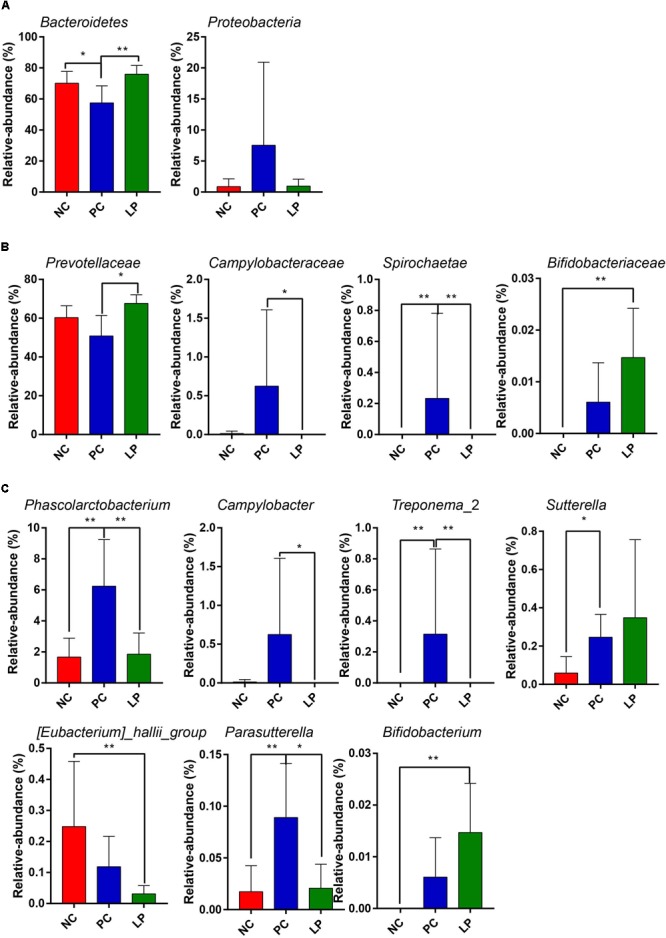
Comparison of the colonic microbial community in weaning piglets from NC, PC, and LP groups. Differences in microbial community at the **(A)** phylum level, **(B)** family level, and **(C)** genus level. Values are expressed as mean ± SD. ^∗^*P* < 0.05, ^∗∗^*P* < 0.01.

**FIGURE 3:**
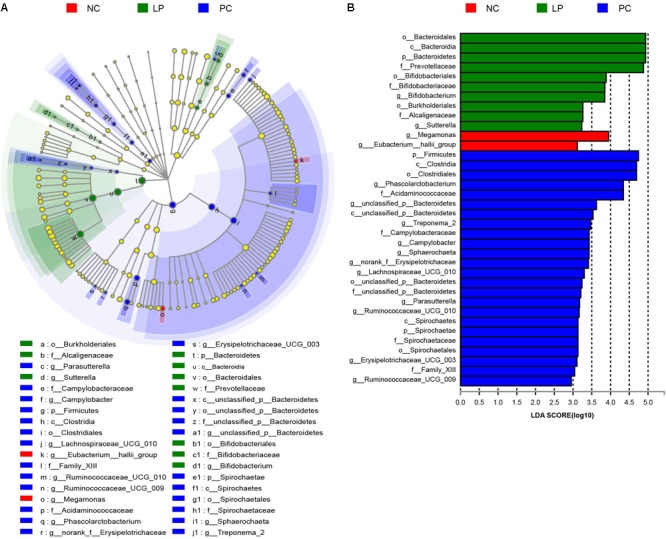
LefSe analysis of colonic microbial community in weaning piglets from NC, PC, and LP groups. **(A)** Cladogram showing microbial species with significant differences in NC, LP, and PC groups. Red, green, and blue indicate different groups, with the species classification at the level of phylum, class, order, family, and genus shown from the inside to outside. The red, green, and blue nodes in the phylogenetic tree represent microbial species that play important roles in the NC, LP, and PC groups, respectively. Yellow nodes represent species with no significant difference. **(B)** Species with significant difference that have an LDA score greater than the estimated value with the default score 3. The length of the histogram represents the LDA score indicating the difference of species in the three groups.

The differences in the microbial communities at the phylum, family, and genus levels are shown in Figure 2A–C. Antibiotics led to a decrease in Bacteroidetes compared to their relative abundance in the NC group (*P* = 0.0497). Bacteroidetes occurred at higher levels in the LP group (75.98%) than in the PC group (57.42%, *P* = 0.0046, Figure 2A and Supplementary Table S1), though there was no significant difference between the LP and NC groups. The relative abundance of Proteobacteria in the PC group (7.54%) was higher than that in the other two groups (NC group: 0.865% and LP group: 0.954%), though there were no significant differences (Figure 2A and Supplementary Table S1). In addition, piglets fed with antibiotics (PC group) had a higher abundance of *Spirochaetae* (0.315%) compared to the NC (0%) and LP groups (0%) (*P* < 0.01, Figure 2A and Supplementary Table S1). We further compared the microbial community at the family level. Antibiotics and probiotics did not differ significantly in their effect on the relative abundance of *Prevotellaceae*; however, the relative abundance was higher in the LP group (67.66%) than in the PC group (50.87%) (*P* = 0.004, Figure 2B and Supplementary Table S2). The relative abundance of *Campylobacteraceae* was higher in the PC group (0.624%) compared to the NC (0.013%) and LP (0%) groups (*P* = 0.034, Figure 2B and Supplementary Table S2). The relative abundance of *Bifidobacteriaceae* was higher in the LP group (0.015%) than in the NC group (0%) (*P* = 0.0047), while there was no significant difference between the PC and NC groups (Figure 2B and Supplementary Table S2). The microbial communities were also compared at the genus level. Antibiotics and probiotics did not have any significant effect on the relative abundance of the *Prevotellaceae* NK3B31 group, the most abundant genus in colonic microbiota, but it trended toward higher abundance in the LP group than in the PC group (*P* = 0.056). The genera *Phascolarctobacterium, Treponema*_2, *Sutterella*, and *Parasutterella* exhibited increased relative abundances in the PC group compared to the NC group (Figure 2C and Supplementary Table S3). The relative abundance of genus *Bifidobacterium* was increased and the genus *Eubacterium_hallii* was decreased in piglets of the LP group compared to the NC group (Figure 2C and Supplementary Table S3). In addition, there was a remarkable increase in the relative abundance of the genus *Campylobacter* in the PC group compared to the LP group. Linear discriminant analysis (LDA) effect size (LefSe) analysis was also performed to confirm the different effects of antibiotics and probiotic on intestinal microbiota in piglets (Figure 3). Interestingly, symbiotic (*Prevotellaceae*) and beneficial (*Bifidobacteriaceae*) bacteria were elevated in the LP group, while harmful bacteria (*Spirochaetae* and *Campylobacteraceae*) were increased in the PC group.

### Comparison of Metabolic Pathway Abundances

**FIGURE 4:**
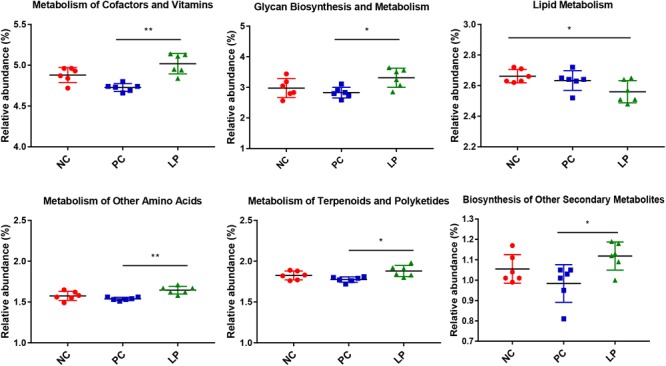
Differences in metabolic functional genes among weaning piglets in NC, PC, and LP groups. ^∗^*P* < 0.05, ^∗∗^*P* < 0.01.

We predicted the microbial metagenome with 16S rRNA gene sequencing using phylogenetic investigation of communities by reconstruction of unobserved states (PICRUSt) (Langille et al., 2013), and found that even with widespread differences in bacterial composition, most functional genes were largely conserved across different groups (Supplementary Table S4). However, the relative abundance of genes related to metabolism was higher in the LP group (49.99%) than in the PC group (48.03%) (*P* < 0.01, Supplementary Table S4). To further study which metabolic genes changed after treatment with probiotics, 40 KEGG Orthology (KO) groups were selected (Supplementary Table S5). We found that, in terms of metabolic pathways, genes that regulated metabolism of cofactors and vitamins, glycan biosynthesis and metabolism, metabolism of other amino acids, metabolism of terpenoids and polyketides, and biosynthesis of other secondary metabolites were more abundant in the LP group than in the PC group (Figure 4). We additionally found that genes related to lipid metabolism were less frequent in the LP group than in the NC group (Figure 4).

### Effects of Probiotics and Antibiotics on SCFA in Colonic Content

To analyze the effects of probiotic *L. plantarum* PFM105 and antibiotics on intestinal microbiota metabolism, we focused on the colonic content of Short-chained fatty acids of weaning piglets. Levels of acetic acid, butyric acid, and total SCFAs were higher in the LP group than in the NC and PC groups (*P* < 0.05, Table 4). However, the acetic and butyric acids and total SCFAs did not differ between the PC and NC groups. There was no significant difference in the colonic content concentrations of propanoic, isobutyric, valeric, and isovaleric acids among the different groups (Table 4). These results demonstrated that *L. plantarum* PFM 105 may promote microbial metabolism and result in increased production of SCFAs (acetic acid and butyric acid), while antibiotics did not have this effect.

**Table 4:** Concentrations of short-chain fatty acids (SCFAs) in the colonic contents of weaning piglets under the different treatments (mg/g dry weight).

Items	NC	PC	LP	*P* values
Acetic acid	1.9 ± 0.67^b^	2 ± 0.69^b^	3.89 ± 1.97^a^	0.0367
Propanoic acid	1.46 ± 0.55	1.54 ± 0.49	2.18 ± 1.05	0.2120
Butyric acid	0.66 ± 0.29^b^	0.67 ± 0.26^b^	1.17 ± 0.32^a^	0.0315
Isobutyric acid	0.07 ± 0.01	0.06 ± 0.02	0.14 ± 0.07	0.0610
Valeric acid	0.05 ± 0.03^ab^	0.03 ± 0.03^b^	0.1 ± 0.06^a^	0.0515
Isovaleric acid	0.11 ± 0.06	0.08 ± 0.09	0.18 ± 0.12	0.2208
Total short-chain fat acids	4.25 ± 1.46^b^	4.38 ± 1.49^b^	7.66 ± 3.22^a^	0.0230

*Different superscript letters within a row represent statistically significant differences among groups (*P* < 0.05).*

### Effects of Probiotics and Antibiotics on Immunoglobulins, Cytokines, and Intestinal Permeability-Related Biomarkers

**FIGURE 5:**
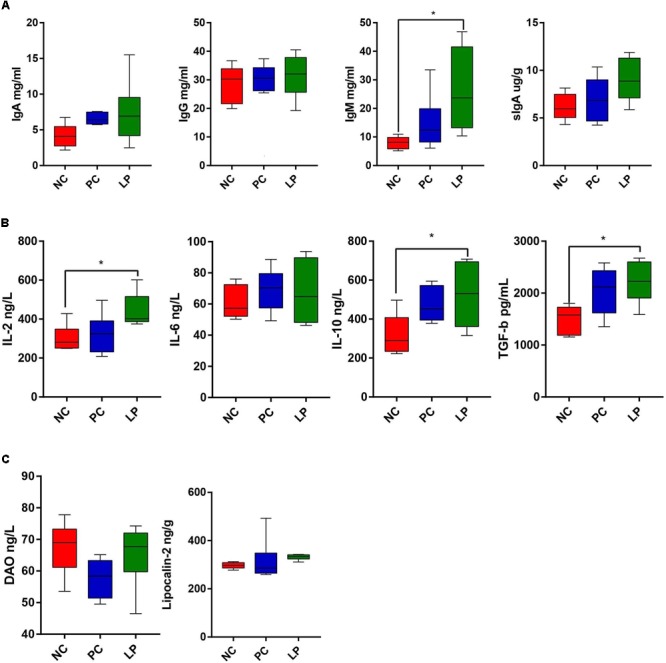
Comparison of serum index among weaning piglets in NC, PC, and LP groups. **(A)** Serum IgG, IgA, IgM, and intestinal SIgA levels among the three groups (*n* = 6). **(B)** Serum cytokines IL-2, IL-6, IL-10, and TGF-β levels among the three groups (*n* = 6). **(C)** Levels of intestinal permeability-related biomarkers DAO and colonic content of Lipocalin-2 among the three groups (*n* = 6). ^∗^*P* < 0.05. IgG, immunoglobulin G; IgA, immunoglobulin A; IgM, immunoglobulin M; sIgA, secretory IgA; TGF-β, transforming growth factor β; IL-2, interleukin 2; IL-6, interleukin 6; IL-10, interleukin 10; DAO, diamine oxidase.

The effects of antibiotics and probiotics on the humoral immunity levels were evaluated by detecting the content of Immunoglobulin G (IgG), Immunoglobulin A (IgA), and Immunoglobulin M (IgM) in serum. The impacts on intestinal immunity were evaluated by detecting the content of secretory IgA (sIgA) in colonic samples (Figure 5A). *L. plantarum* PFM105 treatment significantly increased the total serum IgM (*P* = 0.0447) antibody levels when compared to the NC group (Figure 5A). *L. plantarum* PFM105 treatment also trended toward increasing the intestinal sIgA (*P* = 0.0690) antibody levels as compared to the NC group (Figure 5A). However, the content of serum IgM and intestinal sIgA did not differ between the PC and NC groups. No differences were observed in serum IgG and IgA concentration among the different groups (Figure 5A). We next detected six biomarkers related to gut health of weaning piglets. These biomarkers included a set of serum cytokines [interleukin 2 (IL-2), interleukin 6 (IL-6), interleukin 10 (IL-10), and transforming growth factor β (TGF-β)] as markers for the immune system activation and systemic inflammatory response, serum diamine oxidase (DAO) as a marker for intestinal mucosal integrity, and colonic content of lipocalin-2 as a marker for intestinal inflammation. *L. plantarum* PFM105treatment increased the levels of antibody production mediated IL-2 (*P* = 0.0322), anti-inflammatory mediator IL-10 (*P* = 0.0437), and immune tolerance mediator TGF-β (*P* = 0.0204) over those of the NC group (Figure 5B). However, the levels of IL-2, IL-10, and TGF-β did not differ between the PC and NC groups. Differences in systemic pro-inflammatory cytokines IL-6 (Figure 5B), intestinal permeability marker DAO, and intestinal inflammation marker lipocalin-2 (Figure 5C) were not observed among these groups. These results demonstrated that *L. plantarum* PFM 105, but not antibiotics, may enhance humoral immunity, and prevent intestinal inflammation and excessive systemic immune response, thus promote intestinal health of piglets.

### Alterations in Intestinal Microbiota Composition Were Correlated With Health Parameters

**FIGURE 6:**
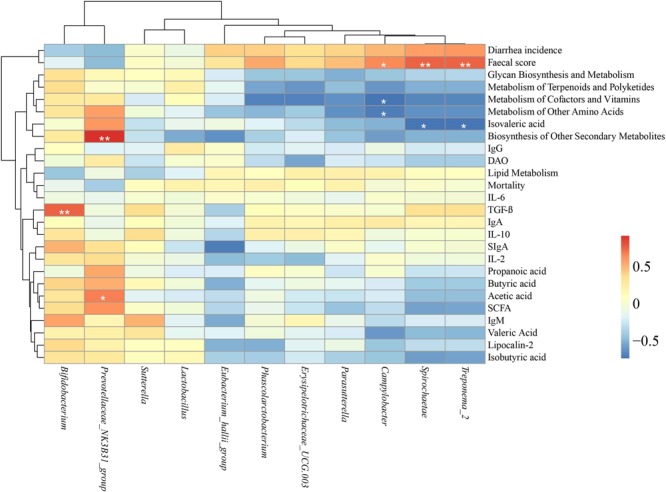
Heatmap of the Spearman rank correlations between the significantly modified microbiota and growth and health parameters in weaning piglets. ^∗^*P* < 0.05, ^∗∗^*P* < 0.01 (following the Spearman correlation analysis). IgG, Immunoglobulin G; IgA, Immunoglobulin A; IgM, Immunoglobulin M; sIgA, secretory IgA; TGF-β, transforming growth factor β; IL-2, interleukin 2; IL-6, interleukin 6; IL-10, interleukin 10; DAO, diamine oxidase; SCFA, short-chained fatty acid.

A Spearman’s rank correlation analysis was performed to evaluate the potential link between alterations in gut microbiota composition and growth and health parameters of weanling piglets (Figure 6). The genus *Bifidobacterium* was positively correlated with increased levels of TGF-β (*P* < 0.01). The genus *Prevotellaceae* NK3B31 group was positively correlated with increased biosynthesis of other secondary metabolites (*P* < 0.05) and acetic acid (*P* < 0.05). The genus *Campylobacter* was positively correlated with fecal score (*P* < 0.05) but negatively correlated with genes of metabolism of cofactors and vitamins and genes of metabolism of other amino acids (*P* < 0.05). The phylum *Spirochaetae* and the genus *Treponema*_2 both were positively correlated with fecal score, and negatively correlated with isovaleric acid (*P* < 0.01).

## Discussion

*Lactobacillus plantarum* PFM105 significantly improved the ADG of piglets as compared to that of the NC group during the third week. Previous studies have shown *L. plantarum* can improve the growth performance of piglets, which aligns with our results (Lee et al., 2012; Suo et al., 2012). Probiotics may improve growth via promoting nutrient absorption by increasing villus height (Suo et al., 2012; Liu et al., 2014). We found that *L. plantarum* PFM105 significantly increased the villus height in the jejunum and trended toward increasing villus height in the ileum, which may be the reason for the increased body weight of these piglets. In this study, antibiotics did not show effects on the development of small intestinal villi or promote growth. Compared to antibiotics, *L. plantarum* PFM105 is likely a better food additive to promote the intestinal development and growth of weaning piglets.

Weaning is usually associated with intestinal disorders as the piglet intestinal microbiota undergoes substantial dynamic changes (Chen et al., 2017). During the experiments, there were two piglets died in the NC group at day 18 and 20, respectively, and a piglet died in the PC group at day 11. The sample size was insufficient to detect differences between groups, but both *L. plantarum* PFM105 and antibiotics could be able to reduce the deaths caused by weaning stress. Previous study showed that piglets fed with *L. plantarum* ZJ316 experienced reduced mortality than those fed with antibiotics, which aligns with our findings (Suo et al., 2012). Additionally, *L. plantarum* PFM105 reduced the diarrhea rate compared to that of the PC group. A previous study also showed *L. plantarum* ZJ316 decreased the diarrhea rate more than antibiotics did (Suo et al., 2012). Other probiotics, such as *Bacillus licheniformis*-*B. subtilis* mixture ameliorated enteritis caused by an enterotoxigenic *E. coli* strain (F4^+^ ETEC) (Zhang et al., 2017). Probiotics, including *L. plantarum* PFM105, may be a more effective means of reducing mortality and diarrhea in weaning piglets.

Piglets treated with *L. plantarum* PFM105 showed an increased Simpson’s diversity index in the gut microbiota compared to that of piglets in the NC group. This could represent a benefit for the weaned animals because of the possible link between the diversity (i.e., degree of simplification) of ecosystems and their ability to respond to perturbations (McCann, 2000; Pieper et al., 2009). Similar results were also reported in previous studies demonstrating that probiotics can increase the Simpson’s diversity index of the microbial ecosystem in piglets (Pieper et al., 2009; Liu et al., 2014). In previous studies, Firmicutes, and Bacteroidetes were the dominant groups in bacterial communities at the phylum level and were not significantly altered by use of probiotics (Wang J. et al., 2018) or of antibiotics (Looft et al., 2012; Holman and Chenier, 2014) in weaning piglets. However, here we found that antibiotics led to a decrease in the relative abundance of Bacteroidetes and an increase in the relative abundance of Proteobacteria and *Spirochaetae*. Representatives of Bacteroidetes provide the host with SCFAs that can supply up to 10% of daily calories through the fermentation of indigestible polysaccharides (McNeil, 1984; Johnson et al., 2017). The anaerobic Proteobacteria are usually associated with an impaired microbiota, or dysbiosis (Litvak et al., 2017). Feeding antibiotics could cause impaired intestinal eubiosis; *E. coli* (phylum Proteobacteria) increased after pigs were treated with antibiotics (Zhang et al., 2018). We also analyzed lower taxonomic levels and found that the relative abundance of *Prevotellaceae* in the LP group was higher than in the PC group. *Prevotellaceae* was the dominant bacteria at the family level (accounting for nearly 60%) in our study, and recent metagenomic studies confirmed the prevalence of *Prevotellaceae* in the cecum, colon, and feces of pigs (Looft et al., 2014). Representatives of *Prevotellaceae* are associated with hemicellulose degradation and are the predominant bacteria in piglets at the nursery stage (Konstantinov et al., 2004). A high *Prevotella* spp. abundance may be essential for post-weaning piglets to be able to digest plant-based diets (Wang J. et al., 2018). High abundance of this bacterium in the LP group suggested that piglets supplemented with *L. plantarum* PFM105 may have strong digestion and absorption capacity.

Opportunistic pathogens, such as *Phascolarctobacterium* (significantly correlated with systemic inflammatory cytokines, such as TNF-α (Ling et al., 2016)), *Campylobacter* (ubiquitous in nature and in domestic animals, but also important in infections in animals) and *Treponema*_2 which belongs to phylum *Spirochaetae* and has been isolated from pig lesions (Svartstrom et al., 2013) were increased after antibiotic treatment compared to the NC group. Certain *Campylobacter* (*C. coli* and *C. hyoilei*) can cause gastrointestinal infection and thus lead to gastroenteritis (Humphrey et al., 2007). *Campylobacter, Treponema*_2, and *Spirochaeta* were positively correlated with fecal score; thus, we hypothesized that these pathogens disturbed the gut microbiome in piglets treated with antibiotics. These opportunistic pathogens were not changed in the LP group when compared to the NC group. By contrast, the well-studied probiotic *Bifidobacterium* increased after *L. plantarum* PFM105 treatment when compared to the NC group, and the relative abundance of *Bifidobacterium* was positively correlated with TGF-β. These beneficial bacteria were not changed in the PC group when compared to the NC group. These results were supported by previous reports indicating that *Bifidobacterium* shows higher anti-inflammatory capacity by inducing intestinal production of IL-10 and TGF-β (Finamore et al., 2012; Herfel et al., 2013). Thus, we hypothesized that *L. plantarum* PFM105 could increase beneficial bacteria, which may assist with energy harvesting and boost anti-inflammatory capacity, while antibiotics could increase pathogenic bacteria, potentially leading to intestinal dysbiosis.

We utilized inferred metagenomics by PICRUSt (Langille et al., 2013) which can reflect the metabolic activities of the microbiota (Peng et al., 2018) to investigate functional differences in the microbiota of piglets in order to determine the metabolic alterations caused by antibiotics or probiotics. We found that even with widespread differences in bacterial community composition, overall function was largely conserved across individuals, and these results were consistent with previous studies in humans (Human Microbiome and Project, 2012). There were no significant differences in metabolic genes between the PC and NC groups. However, metabolic genes were more abundant in the LP group compared to PC group, implying that microbial metabolism tended to be more vigorous after treatment with *L. plantarum* PFM105. We found that genes of cofactors and vitamins metabolism and glycan biosynthesis and metabolism were more abundant in the LP group than in the PC group. Previous studies have shown that vitamins and cofactors, especially of the vitamin B family, are critical for the bioconversion of nutrients to energy and for maintaining homeostasis (McDonald, 2009; Hu et al., 2016). The glycan biosynthesis and metabolism genes are important for carbohydrate metabolism (Hu et al., 2016). These results indicated that *L. plantarum* PFM105 may promote energy metabolism. Previous studies have also shown that these genes were more abundant in aged pigs (Hu et al., 2016). Gao et al. (2017) found that the maturation of intestinal microbiota was greatly accelerated by probiotic (*L. plantarum* LP-8) feeding, yet significantly delayed by antibiotic feeding. Probiotic *L. plantarum* PFM105 might accelerate intestinal microbiota maturation by increasing important metabolic genes.

Short-chained fatty acids are important for gut integrity, glucose homeostasis, and immune function (Morrison and Preston, 2016). They are produced by the colonic anaerobic microbial community through fermenting indigestible fiber matter and some luminal amino acids (Blachier et al., 2007; Kong et al., 2016). In our study, acetic and propanoic acids were the major SCFAs produced in the colon, which was consistent with previous findings in the colon of pregnant Huanjiang mini-pigs (Kong et al., 2016) and in the feces of primiparous sows (Paßlack et al., 2015). In the current study, the levels of these SCFAs were higher in the LP group than in the NC and PC groups. Acetic acid is reported to inhibit pathogenic bacteria, and butyric acid acts as a major energy source for colonic epithelial cells (Morrison and Preston, 2016). The increasing quantity of butyric acid in colonic content after the administration of *L. plantarum* PFM105 is consistent with previous findings in pigs administrated with *Lactobacillus reuteri* I5007 (Liu et al., 2014, 2017). The increased content of acetic acid and butyric acid in the LP group might due to the increased proportion of family *Prevotellaceae* which is known to be important for polysaccharide degradation and SCFAs formation. All identified enzymes involved in polysaccharide (starch) degradation are associated with *Prevotellaceae* (Ivarsson et al., 2014; Heinritz et al., 2016). Our results showed that the *Prevotellaceae* NK3B31 group was positively correlated with acetic acid, which was also demonstrated in a recent study in piglets administrated with *L. plantarum* ZLP001 (Wang J. et al., 2018). Though the unique interaction between *L. plantarum* and *Prevotellaceae* is still unclear, we hypothesize that *L. plantarum* PFM105 might promote the production of SCFAs by increasing *Prevotellaceae*.

Immunoglobulin G, Immunoglobulin A, and Immunoglobulin M represent the main antibody isotype found in blood and extracellular fluid, the predominant immunoglobulin isotype expressed in mucosal tissues, and the major component of natural antibodies, respectively. They are the main immunoglobulins involved in humoral immunity (Luo et al., 2013). In our study, the expression level of IgM was increased in LP group piglets compared to NC group piglets, indicating that the humoral immunity level of piglets was improved by *L. plantarum* PFM105. Zhu et al. (2017) found that feeding pigs a diet containing probiotics could increase serum IgM levels, which supports our findings. Considering recent evidence suggesting that microbiota can modulate intestinal barrier integrity, and improve immunology tolerance of newborn individuals (Huang et al., 2015; Cheng et al., 2018), we further assessed the gut permeability and intestinal or systemic inflammatory response of piglets. Cytokines play a crucial role in immune and inflammatory responses, and their balance is important for protection against infection. Cytokine IL-2 is critical for regulating lymphoid homeostasis (Ma et al., 2006). IL-10, an anti-inflammatory cytokine, can prevent over-activation of the immune response and suppress the production of pro-inflammatory cytokines and thus plays an integral role in maintenance of immune homeostasis (Opal and Depalo, 2004). TGF-β exerts systemic immune suppression and inhibits host immunosurveillance (Yang et al., 2010). In the present study, we found that the expression levels of IL-2, IL-10, and TGF-β were increased in the LP group over those of the NC group. We hypothesized that the increased pro-inflammatory and anti-inflammatory cytokines caused by *L. plantarum* PFM105 treatment may have decreased susceptibility to pathogenic infection. Previous studies showed that probiotic *Bifidobacterium* can increase serum TGF-β levels (Ouwehand et al., 2008) and probiotic *L. plantarum* enhance IL10 production (Bosch et al., 2012; Noguchi et al., 2012), which also supports our results.

## Conclusion

Weaning represents a major challenge to a developing pig acclimating to gastrointestinal microbial colonization, and is often associated with gastrointestinal disorders. In pig husbandry, antibiotics are commonly used to alleviate weaning stress, but our study found that the use of probiotic *L. plantarum* PFM 105 may be more effective. *L. plantarum* PFM 105 could strongly improve the development of small intestinal villi and the growth performance of weaning piglets. Compared to antibiotics, *L. plantarum* PFM 105 enhanced piglet clinical performance, reduced mortality, and lowered incidence of diarrhea. *L. plantarum* PFM 105 might also enhance humoral immunity, prevent intestinal inflammation, and avert excessive systemic immune response. *L. plantarum* PFM 105 modulated the piglet gut microbiota by increasing the abundance of symbiotic and beneficial bacteria (e.g., *Prevotellaceae* and *Bifidobacteriaceae*), while antibiotics increased the occurrence of harmful bacteria (e.g., *Spirochaetae* and *Campylobacteraceae*). *L. plantarum* PFM 105 increased the expression levels of genes related to metabolism of cofactors and vitamins, and to glycan biosynthesis and metabolism, which may enhance the metabolic capacity of the microbiota in piglets. Our results demonstrate the possibility of using *L. plantarum* PFM 105 instead of antibiotics to promote intestinal development and modulate gut microbiota in weaning piglets.

## Materials and Methods

### Statement of Ethics for the Care and Use of Animals

The experimental procedures used in this study were approved by the Laboratory Animal Ethical Commission of the Chinese Academy of Sciences and performed according to its guidelines. Humane animal care was practiced throughout the trial and every effort was made to minimize suffering for piglets.

### Animals, Diets and Sampling

A total of 144 normal weaning piglets (72 males and 72 females) from 28 litters (Landrace × Large White, 28 days of age, 8.22 ± 0.38 kg) were obtained from the LongDa Foodstuff Group Co., Ltd (Shandong Province, China) and were allocated randomly to three groups for the 21 days trial, balancing for litter and gender. The negative control (NC) group was fed the base diet without any antibiotics or probiotics. The positive control (PC) group was fed the base diet plus the antibiotic apramycin sulfate at 25 mg/kg BW in feed. The probiotic (LP) group was fed the base diet plus the probiotic strain *L. plantarum* PFM105 (CGMCC 16113, isolated from healthy sow intestine) in feed. Prior to the start of the trial, no clinical signs of diarrhea or other diseases were observed in any of the piglets. All pigs in this study were selected from one delivery room and had similar genetic backgrounds and husbandry practices. Each group included 48 piglets in six replicates (8 piglets in each replicate and housed in a pen). These 18 pens were in the same nursing house. Room temperature was maintained at 26°C, and the humidity was maintained constant at 65–75%. All pigs were fed four times a day with customized corn-soybean feed (free of probiotics and antibiotics) containing 19% crude protein, and details are provided in the supplementary material for ingredients and nutrient composition (Supplementary Table S6) (Yin et al., 2017). Water was available *ad libitum* from nipple drinkers. Lyophilized LP was provided by the Center for Technology Transfer and Transformation of Institute of Microbiology, Chinese Academy of Sciences (Beijing, China) and was added to the feed for piglets at a final concentration of 2 × 10^7^ CFU/g. To ensure dose accuracy, the concentration of live bacteria in the powder was verified based on culture-based counting. Moreover, to verify the purity of the probiotic preparation, 10 clones were randomly picked from a de Man, Rogosa and Sharpe (MRS) plate derived from the bacterial freeze-dried powder. Genomic DNA was extracted from each of the clone and 16S genes were amplified via a universal bacterial 16S PCR primer (27F/1492R, listed in Supplementary Table S7) and sequenced by Beijing Ruiboxingke Biotechnology Co. Ltd (Beijing, China). All 10 clones were confirmed as *L. plantarum*, which validated the purity of our probiotic preparation.

All piglets in each pen were weighed individually at days 0, 14, and 21 during the trial. The feed consumed by each pen was monitored daily. ADG, ADFI, and feed conversion ratio (FCR; feed consumed/weight gain) were calculated for the periods of 1–14, 14–21, and 1–21 days. The health status of piglets during the experiments was assessed by fecal consistency scoring using a four-grade system, where 0 corresponded to firm and dry, 1 to pasty, 2 to thick and fluid, and 3 to watery (De Cupere et al., 1992). The fecal score was calculated as the sum of the diarrhea score over the period divided by the number of piglet days in the period. The occurrence of diarrhea was defined as maintaining a score of 3 for 1 day (Liu et al., 2017). The incidence of diarrhea (%) was calculated as the sum of the total number of diarrheal piglets over the period divided by the number of piglet days in the period multiplied by 100. The mortality (%) was calculated as the sum of the total number of dead piglets over the period divided by the number of piglets multiplied by 100.

On day 21, one median-weight piglet from each replicate from different pens was sacrificed and 10 ml of blood was collected. The luminal content samples were collected at the same site for each gut location. Briefly, after opening the visceral cavity, esophagus and rectum were clamped to avoid spilling of gastrointestinal digesta and thus contamination of other intestinal parts. Immediately after removing the gastrointestinal tract (GIT) from the visceral cavity, the mid-jejunum, mid-ileum, mid-colon, and rectum were separated by clamping to avoid mixing of digesta from adjacent segments of the GIT. The luminal contents were separately gathered from the middle section of the colon. Subsequently, intestinal segments were disclosed at the mesentery with sterile instruments and digesta was removed. The experimental platform was disinfected before each sample was collected to avoid cross-contamination between samples. All samples were harvested within 30 min of slaughtering and transferred immediately to liquid nitrogen for temporary storage. Samples were then sent to the laboratory where they were stored at -80°C until analysis.

### Detection of Immunoglobulin, Cytokines, and DAO in Serum and of Intestinal SIgA and Lipocalin-2 in Colonic Contents

Serum was collected and centrifuged at 4000 rpm for 10 min at 4°C before being stored at -80°C until IgG, IgA, and IgM were quantified. Serum total IgG and IgA were detected by porcine enzyme-linked immunosorbent assay (ELISA) kits (Nanjing Jiancheng Bioengineering Institute, Nanjing, China). Colonic content (1 g) was collected and mixed with an equal volume of PBS and centrifuged at 1000 rpm for 15 min. The supernatant was then stored at -80 °C until sIgA was quantified by porcine ELISA kits (Jiangsu Meimian Industry Co., Ltd, Nanjing, China). IL-2, IL-6, IL-10, TGF-β, DAO, and lipocalin-2 concentrations were determined using porcine ELISA kits according to the manufacturer’s instructions (Jiangsu Bo Deep Biological Technology Co., Ltd., Nanjing, China). Their concentrations were then calculated from the standard curves. All procedures were performed with 3 repetitions.

### Quantification of SCFAs in Colonic Content Samples

Short-chained fatty acids including acetic acid, propanoic acid, butyric acid, isobutyric acid, valeric acid, and isovaleric acid were analyzed as described previously with minor modifications (Wang K. et al., 2018; Yin et al., 2018). Colonic contents were collected from the middle segment of the colon from piglets after 21 days of treatment. Each sample was lyophilized and then pestled using a mortar. 100 mg of the homogenic powders was extracted with 1 mL of methanol (gradient grade for liquid chromatography LiChrosolv^®^ Reag. Ph Eur EA). After 10 min sonication, the samples were centrifuged (6000rpm for 10 min), and the supernatants were used for GC-MS analysis. GC-MS was performed on a GC-MS-QP2010 Ultra with an autosampler (SHIMADZU) and the Rtx-wax capillary column (30 m, 0.25 mm i.d., 0.25 μm film thickness; SHIMADZU). Oven temperature was programmed from 60 to 100°C at 5°C/min, with a 1 min hold; to 150°C at 5 °C/min, with a 5 min hold; to 225°C at 30°C/min, with a 20 min hold. Injection of a 2 μL sample was performed at 230°C. Helium, at a flow of 1.2 mL/min, was the carrier gas. Electronic impact was recorded at 70 eV.

### Genomic DNA Extraction and 16S rRNA Gene Sequencing

Colonic content DNA was extracted from 0.2 g of sample using the protocol of the QIAamp PowerFecal DNAKit (Qiagen, Dusseldorf, Germany). DNA was eluted in ddH_2_O and stored at -80°C until use. The V3-V4 hypervariable region of the 16S rDNA gene was targeted and the primers used are listed in Supplementary Table S7. The bacteria 16S ribosomal RNA gene was amplified. PCR reactions were performed in triplicate in a 20 μL mixture containing 4 μL of 5 × FastPfu Buffer, 2 μL of 2.5 mM dNTPs, 0.8 μL of each primer (5 μM), 0.4 μL of FastPfu Polymerase, and 10 ng of template DNA. Amplicons were extracted from 2% agarose gels and purified using the AxyPrep DNA Gel Extraction Kit (Axygen Biosciences, Union City, CA, United States) according to the manufacturer’s instructions and quantified using QuantiFluor^TM^ -ST (Promega, Madison, WI, United States). Purified amplicons were pooled in equimolar concentrations and paired-end sequenced (2 × 250) on an Illumina MiSeq platform according to the standard protocols.

### Bioinformatics Analysis

Paired-end reads were merged using FLASH (Magoc and Salzberg, 2011), which was designed to merge paired-end reads when at least some of the reads overlap the read generated from the opposite end of the same DNA fragment, and the splicing sequences were called raw tags. Quality filtering on the raw tags were performed under specific filtering conditions to obtain the high-quality clean tags according to the QIIME quality-control process (Bokulich et al., 2013). The tags were compared with the reference database (Gold database) using the UCHIME algorithm to detect chimera sequences which were later removed (Edgar et al., 2011; Haas et al., 2011). OTUs were clustered with a 97% similarity cutoff using UPARSE (Edgar, 2013) and chimeric sequences were identified and removed using UCHIME. The taxonomy of each 16S rRNA gene sequence was analyzed by RDP Classifier against the SILVA (SSU115) 16S rRNA database using a confidence threshold of 70% (DeSantis et al., 2006; Wang et al., 2007). Sequences with higher than 97% similarity were assigned to the same OTU. A representative sequence for each OTU was screened for further annotation. OTU abundance information was normalized using a standard of sequence number corresponding to the sample with the fewest sequences. Subsequent analysis of alpha diversity was performed based on this output normalized data. Alpha diversity was analyzed through six indices, including observed-species (OS), Chao1, Shannon, Simpson, ACE, and Good-coverage. β-diversity was analyzed by PCA was conducted based on phylum and OTU, and the hierarchical clustering tree was constructed based on Unweight Unifrac distances. All of these indices in our samples were calculated with QIIME (Version 1.7.0) and displayed with R software (Version 2.15.3). The dominant bacterial community difference between groups was detected using LDA effect size (LefSe). The biomarkers used in the present study had an effect-size threshold of three.

### Morphological Analyses

Piglet jejunums and ileum were prepared using our previous described methods (Wang T. et al., 2018). Briefly, they were fixed with 10% paraformaldehyde-PBS overnight and then dehydrated and embedded in paraffin blocks. A 5 μm section was cut, deparaffinized, hydrated, and then stained with hematoxylin and eosin (H&E). Villus length and crypt depth of at least three villi or crypts per slide were measured using Image-Pro Plus software 6. Six piglets were studied from each group. The data collectors were unaware of the treatment status of the examined slides.

### Statistical Analysis

Data shown are means ± standard deviation (SD) or standard error of the mean (SEM). Data were analyzed by one-way ANOVA followed by Dunnett multiple comparisons (Prism 7.0) if the data were in Gaussian distribution and had equal variance or analyzed by the Kruskal-Wallis test followed by Dunn’s multiple comparisons (Prism 7.0) if the data were not normally distributed. The Gaussian distribution of data was analyzed by the Kolmogorov-Smirnov test (Prism 7.0). The variance of data was analyzed by homogeneity of variance test (SPSS 19.0) or the Brown-Forsythe test (Prism 7.0). Differences with *P* < 0.05 were considered significant. Statistical evaluation of the incidence of diarrhea was performed using Pearson’s chi-square test. The non-parametric Friedman’s test using procedure FREQ was carried out to compare non-normally distributed and repeated-measure diarrhea scores. Correlations were analyzed by using Spearman’s correlation in R 3.4.4 (The R Foundation) with the RStudio psych package and pheatmap for the heat map. Correlation results were corrected by FDR analysis according to the Benjamini-Hochberg procedure, with an α of <0.05.

## Author Contributions

JinZ, KT, and TW designed the study. TW and KT wrote the manuscript. TW, YL, JieZ, ED, WS, and MZ performed the experiments. YT and JinZ edited the manuscript. All authors have discussed the results and reviewed the manuscript.

## Conflict of Interest Statement

XZ and ED were employed by company LongDa Foodstuff Group Co., Ltd. The remaining authors declare that the research was conducted in the absence of any commercial or financial relationships that could be construed as a potential conflict of interest.
