# Supplementary material for: Lactobacillus plantarum PFM 105 Promotes Intestinal Development Through Modulation of Gut Microbiota in Weaning Piglets
Source: Front Microbiol. 2019 Feb 5;10:90. doi: 10.3389/fmicb.2019.00090 (PMC6371750; doi:10.3389/fmicb.2019.00090)
Supplement: Supplementary file 1 [file Table_1.DOCX]

***Lactobacillus plantarum* PFM 105 promotes intestinal development through modulation of gut microbiota** **in weaning piglets**

**Tianwei Wang^1,2^†, Kunling Teng^1^†, Yayong Liu^1,2^, Weixiong Shi^1,2^, Jie Zhang^1^, Enqiu Dong^3^, Xin Zhang^3^, Yong Tao^1,2^, Jin Zhong^1,2*^**

^1^ State Key Laboratory of Microbial Resources, Institute of Microbiology, Chinese Academy of Sciences, Beijing, China

^2^ University of Chinese Academy of Sciences, Beijing, China

^3^ LongDa Foodstuff Group Co., Ltd, Shandong Province, China

***Correspondence:**

Jin Zhong

[zhongj@im.ac.cn](mailto:zhongj@im.ac.cn)

Table S1. Relative abundance (%) of bacterial phyla in the colonic microbiota of piglets in different groups, determined by Illumina sequencing of 16S rRNA tags.

| **Taxa** | **Groups** | | |  |
| --- | --- | --- | --- | --- |
| **Phylum** | **NC**  **Mean (%)** | **PC**  **Mean (%)** | **LP**  **Mean (%)** | ***P*-value** |
| *Bacteroidetes* | 70.110 | 57.420 | 75.980 | 0.011 |
| *Firmicutes* | 28.840 | 34.160 | 22.910 | 0.055 |
| *Proteobacteria* | 0.865 | 7.540 | 0.954 | 0.596 |
| *Tenericutes* | 0.061 | 0.326 | 0.067 | 0.688 |
| *Actinobacteria* | 0.113 | 0.210 | 0.073 | 0.688 |
| *Spirochaetae* | ND | 0.315 | ND | 0.002 |
| *Cyanobacteria* | 0.008 | 0.017 | 0.019 | 0.772 |
| *Fibrobacteres* | ND | 0.009 | ND | 0.613 |
| *Fusobacteria* | 0.003 | ND | 0.002 | 0.596 |
| *Deferribacteres* | 0.001 | 0.001 | ND | 0.688 |

“ND”, not detected.
