# Supplementary material for: Lactobacillus plantarum PFM 105 Promotes Intestinal Development Through Modulation of Gut Microbiota in Weaning Piglets
Source: Front Microbiol. 2019 Feb 5;10:90. doi: 10.3389/fmicb.2019.00090 (PMC6371750; doi:10.3389/fmicb.2019.00090)
Supplement: Supplementary file 2 [file Table_2.DOCX]

***Lactobacillus plantarum* PFM 105 promotes intestinal development through modulation of gut microbiota** **in weaning piglets**

**Tianwei Wang^1,2^†, Kunling Teng^1^†, Yayong Liu^1,2^, Weixiong Shi^1,2^, Jie Zhang^1^, Enqiu Dong^3^, Xin Zhang^3^, Yong Tao^1,2^, Jin Zhong^1,2*^**

^1^ State Key Laboratory of Microbial Resources, Institute of Microbiology, Chinese Academy of Sciences, Beijing, China

^2^ University of Chinese Academy of Sciences, Beijing, China

^3^ LongDa Foodstuff Group Co., Ltd, Shandong Province, China

***Correspondence:**

Jin Zhong

[zhongj@im.ac.cn](mailto:zhongj@im.ac.cn)

Table S2. Relative abundance (%) of bacterial families in the colonic microbiota of piglets in different groups, determined by Illumina sequencing of 16S rRNA tags.

| **Taxa** | **Groups** | | |  |
| --- | --- | --- | --- | --- |
| **Family** | **NC**  **Mean (%)** | **PC**  **Mean (%)** | **LP**  **Mean (%)** | ***P* value** |
| Prevotellaceae | 60.300 | 50.870 | 67.660 | 0.007 |
| Lachnospiraceae | 10.700 | 13.050 | 6.633 | 0.293 |
| Ruminococcaceae | 9.692 | 12.110 | 8.049 | 0.548 |
| Bacteroidaceae | 3.960 | 2.294 | 4.277 | 0.880 |
| Acidaminococcaceae | 1.748 | 6.268 | 1.864 | 0.124 |
| Veillonellaceae | 2.557 | 0.423 | 4.071 | 0.293 |
| Porphyromonadaceae | 3.366 | 1.012 | 1.541 | 0.879 |
| Bacteroidales S24-7 | 1.812 | 1.656 | 1.852 | 0.917 |
| Neisseriaceae | 0.559 | 3.957 | 0.001 | 0.642 |
| Lactobacillaceae | 2.003 | 0.475 | 0.569 | 0.668 |
| Enterobacteriaceae | 0.090 | 2.131 | 0.298 | 0.929 |
| Clostridiaceae | 0.788 | 0.815 | 0.911 | 0.880 |
| Rikenellaceae | 0.585 | 1.202 | 0.533 | 0.649 |
| Peptostreptococcaceae | 0.986 | 0.334 | 0.504 | 0.731 |
| Erysipelotrichaceae | 0.270 | 0.431 | 0.192 | 0.548 |
| Alcaligenaceae | 0.076 | 0.335 | 0.369 | 0.118 |
| Campylobacteraceae | 0.013 | 0.624 | ND | 0.029 |
| norank Mollicutes RF9 | 0.061 | 0.319 | 0.067 | 0.719 |
| Pasteurellaceae | 0.108 | 0.018 | 0.278 | 0.642 |
| Coriobacteriaceae | 0.109 | 0.201 | 0.056 | 0.642 |
| unclassified Bacteroidales | 0.014 | 0.242 | 0.092 | 0.474 |
| Spirochaetaceae | ND | 0.315 | ND | 0.002 |
| Helicobacteraceae | 0.005 | 0.239 | 0.008 | 0.744 |
| Family XIII | 0.030 | 0.139 | 0.048 | 0.172 |
| Desulfovibrionaceae | 0.003 | 0.213 | ND | 0.642 |
| norank Bacteroidale | 0.068 | 0.046 | 0.022 | 0.718 |
| Streptococcaceae | 0.054 | 0.025 | 0.040 | 0.929 |
| unclassified Bacteroidetes | ND | 0.102 | ND | 0.181 |
| Clostridiales vadinBB60 | 0.005 | 0.058 | 0.021 | 0.642 |
| norank Gastranaerophilales | 0.003 | 0.013 | 0.017 | 0.917 |
| Succinivibrionaceae | 0.010 | 0.022 | ND | 0.474 |
| Christensenellaceae | 0.003 | 0.016 | 0.003 | 0.253 |
| Bifidobacteriaceae | ND | 0.006 | 0.015 | 0.007 |
| Peptococcaceae | 0.003 | 0.015 | ND | 0.642 |
| Defluviitaleaceae | 0.003 | 0.007 | 0.003 | 0.700 |
| norank Cyanobacteri | 0.004 | 0.004 | 0.002 | 0.700 |
| Streptomycetaceae | 0.004 | 0.003 | 0.003 | 0.879 |
| Fibrobacteraceae | ND | 0.009 | ND | 0.642 |
| Anaeroplasmataceae | ND | 0.007 | ND | 0.642 |
| Leptotrichiaceae | 0.003 | ND | 0.002 | 0.642 |
| Deferribacteraceae | 0.001 | 0.001 | ND | 0.744 |
| unclassified Clostridiales | ND | ND | 0.002 | 0.642 |

“ND”, not detected.
