# Supplementary material for: Lactobacillus plantarum PFM 105 Promotes Intestinal Development Through Modulation of Gut Microbiota in Weaning Piglets
Source: Front Microbiol. 2019 Feb 5;10:90. doi: 10.3389/fmicb.2019.00090 (PMC6371750; doi:10.3389/fmicb.2019.00090)
Supplement: Supplementary file 3 [file Table_3.DOCX]

***Lactobacillus plantarum* PFM 105 promotes intestinal development through modulation of gut microbiota** **in weaning piglets**

**Tianwei Wang^1,2^†, Kunling Teng^1^†, Yayong Liu^1,2^, Weixiong Shi^1,2^, Jie Zhang^1^, Enqiu Dong^3^, Xin Zhang^3^, Yong Tao^1,2^, Jin Zhong^1,2*^**

^1^ State Key Laboratory of Microbial Resources, Institute of Microbiology, Chinese Academy of Sciences, Beijing, China

^2^ University of Chinese Academy of Sciences, Beijing, China

^3^ LongDa Foodstuff Group Co., Ltd, Shandong Province, China

***Correspondence:**

Jin Zhong

[zhongj@im.ac.cn](mailto:zhongj@im.ac.cn)

Table S3. Relative abundance (%) of bacterial genera in the colonic microbiota of piglets in different groups, determined by Illumina sequencing of 16S rRNA tags.

| **Taxa** | **Groups** | | |  |
| --- | --- | --- | --- | --- |
| **Genera** | **NC**  **Mean (%)** | **PC**  **Mean (%)** | **LP**  **Mean (%)** | **P value** |
| *Prevotella_9* | 22.100 | 21.680 | 17.790 | 0.873 |
| *Prevotellaceae_NK3B31_group* | 16.520 | 10.970 | 33.570 | 0.057 |
| *unclassified_f__Lachnospiraceae* | 6.230 | 5.525 | 3.330 | 0.709 |
| *unclassified_f__Prevotellaceae* | 5.985 | 3.398 | 4.793 | 0.919 |
| *Alloprevotella* | 5.644 | 2.908 | 3.355 | 0.517 |
| *Bacteroides* | 3.960 | 2.294 | 4.277 | 0.894 |
| *Phascolarctobacterium* | 1.671 | 6.235 | 1.854 | 0.015 |
| *Prevotella_1* | 2.212 | 4.366 | 2.578 | 0.873 |
| *norank_f__Prevotellaceae* | 3.615 | 2.837 | 1.720 | 0.873 |
| *Faecalibacterium* | 3.341 | 2.570 | 1.854 | 0.785 |
| *Prevotella_2* | 1.952 | 2.266 | 1.896 | 0.873 |
| *Parabacteroides* | 3.364 | 0.977 | 1.541 | 0.873 |
| *norank_f__Bacteroidales_S24-7_group* | 1.812 | 1.656 | 1.852 | 0.933 |
| *Prevotellaceae_UCG-003* | 2.007 | 1.152 | 1.733 | 0.971 |
| *Lachnospiraceae_NK4A136_group* | 1.085 | 2.843 | 0.732 | 0.814 |
| *Subdoligranulum* | 1.120 | 1.790 | 1.682 | 0.982 |
| *Leeia* | 0.559 | 3.957 | 0.001 | 0.712 |
| *Megamonas* | 2.146 | 0.071 | 2.145 | 0.454 |
| *unclassified_f__Ruminococcaceae* | 1.432 | 1.318 | 0.923 | 0.873 |
| *Ruminiclostridium_9* | 0.890 | 1.070 | 1.153 | 0.848 |
| *Lactobacillus* | 2.003 | 0.475 | 0.569 | 0.755 |
| *Escherichia-Shigella* | 0.090 | 2.131 | 0.298 | 0.971 |
| *Clostridium_sensu_stricto_1* | 0.747 | 0.685 | 0.879 | 0.873 |
| *Megasphaera* | 0.345 | 0.118 | 1.612 | 0.990 |
| *Rikenellaceae_RC9_gut_group* | 0.375 | 1.152 | 0.365 | 0.709 |
| *Blautia* | 0.549 | 0.846 | 0.297 | 0.517 |
| *Oscillospira* | 0.221 | 0.655 | 0.572 | 0.627 |
| *Coprococcus_3* | 0.309 | 0.776 | 0.271 | 0.814 |
| *norank_f__Ruminococcaceae* | 0.533 | 0.375 | 0.315 | 0.866 |
| *Ruminococcaceae_UCG-014* | 0.426 | 0.690 | 0.094 | 0.848 |
| *Ruminococcaceae_UCG-005* | 0.348 | 0.552 | 0.227 | 0.709 |
| *Intestinibacter* | 0.585 | 0.207 | 0.268 | 0.891 |
| *Lachnoclostridium* | 0.182 | 0.271 | 0.559 | 0.517 |
| *Prevotella_7* | ND | 0.992 | 0.003 | 0.709 |
| *[Eubacterium]_eligens_group* | 0.346 | 0.368 | 0.264 | 0.873 |
| *Ruminococcaceae_UCG-002* | 0.091 | 0.717 | 0.126 | 0.494 |
| *Anaerotruncus* | 0.226 | 0.475 | 0.216 | 0.517 |
| *Terrisporobacter* | 0.400 | 0.127 | 0.236 | 0.873 |
| *[Eubacterium]_coprostanoligenes_*  *group* | 0.179 | 0.463 | 0.078 | 0.509 |
| *Prevotellaceae_UCG-001* | 0.252 | 0.257 | 0.171 | 0.866 |
| *Oscillibacter* | 0.088 | 0.245 | 0.333 | 0.517 |
| *Sutterella* | 0.059 | 0.246 | 0.348 | 0.025 |
| *Campylobacter* | 0.013 | 0.624 | ND | 0.029 |
| *[Ruminococcus]_torques_group* | 0.374 | 0.115 | 0.135 | 0.812 |
| *Lachnospiraceae_UCG-004* | 0.073 | 0.157 | 0.374 | 0.709 |
| *Roseburia* | 0.346 | 0.112 | 0.053 | 0.712 |
| *[Ruminococcus]_gauvreauii_group* | 0.107 | 0.221 | 0.163 | 0.582 |
| *Butyricicoccus* | 0.148 | 0.174 | 0.166 | 0.990 |
| *norank_o__Mollicutes_RF9* | 0.061 | 0.319 | 0.067 | 0.814 |
| *Ruminococcaceae_UCG-008* | 0.165 | 0.211 | 0.048 | 0.494 |
| *Alistipes* | 0.206 | 0.035 | 0.168 | 0.873 |
| *Actinobacillus* | 0.108 | 0.018 | 0.278 | 0.709 |
| *norank_f__Lachnospiraceae* | 0.107 | 0.189 | 0.106 | 0.455 |
| *[Eubacterium]_hallii_group* | 0.248 | 0.118 | 0.031 | 0.029 |
| *Lachnospira* | 0.133 | 0.237 | 0.024 | 0.636 |
| *unclassified_o__Bacteroidales* | 0.014 | 0.242 | 0.092 | 0.517 |
| *Anaerovibrio* | 0.042 | 0.046 | 0.260 | 0.940 |
| *Pseudoflavonifractor* | 0.220 | 0.064 | 0.054 | 0.984 |
| *Catenibacterium* | 0.083 | 0.099 | 0.113 | 0.755 |
| *Lachnospiraceae_UCG-010* | 0.021 | 0.249 | 0.022 | 0.405 |
| *Holdemanella* | 0.082 | 0.137 | 0.048 | 0.894 |
| *Fusicatenibacter* | 0.044 | 0.185 | 0.032 | 0.873 |
| *Pseudobutyrivibrio* | 0.109 | 0.062 | 0.086 | 0.755 |
| *Dorea* | 0.122 | 0.125 | 0.007 | 0.517 |
| *Helicobacter* | 0.005 | 0.239 | 0.008 | 0.858 |
| *Ruminococcaceae_NK4A214_group* | 0.062 | 0.142 | 0.042 | 0.517 |
| *Lachnospiraceae_UCG-007* | 0.019 | 0.196 | 0.021 | 0.971 |
| *Collinsella* | 0.049 | 0.150 | 0.035 | 0.785 |
| *Treponema_2* | ND | 0.233 | ND | 0.002 |
| *Coprococcus_1* | 0.125 | 0.067 | 0.035 | 0.627 |
| *Desulfovibrio* | 0.003 | 0.213 | ND | 0.709 |
| *Clostridium_sensu_stricto_6* | 0.041 | 0.131 | 0.032 | 0.894 |
| *Ruminiclostridium_5* | 0.067 | 0.054 | 0.070 | 0.982 |
| *Mitsuokella* | 0.004 | 0.163 | 0.020 | 0.894 |
| *Ruminococcus_1* | 0.009 | 0.153 | ND | 0.517 |
| *Ruminococcus_2* | 0.020 | 0.107 | 0.024 | 0.709 |
| *Family_XIII_AD3011_group* | 0.023 | 0.098 | 0.030 | 0.494 |
| *Asteroleplasma* | 0.080 | 0.067 | 0.001 | 0.873 |
| *norank_o__Bacteroidales* | 0.068 | 0.046 | 0.022 | 0.812 |
| *Parasutterella* | 0.017 | 0.089 | 0.021 | 0.025 |
| *Acidaminococcus* | 0.077 | 0.033 | 0.010 | 0.709 |
| *Streptococcus* | 0.054 | 0.025 | 0.040 | 0.971 |
| *[Eubacterium]_fissicatena_group* | 0.007 | 0.066 | 0.032 | 0.709 |
| *[Eubacterium]_xylanophilum_group* | 0.072 | 0.033 | ND | 0.517 |
| *unclassified_p__Bacteroidetes* | ND | 0.102 | ND | 0.405 |
| *Ruminococcaceae_UCG-013* | 0.043 | 0.033 | 0.022 | 0.869 |
| *Ruminococcaceae_UCG-003* | 0.013 | 0.055 | 0.029 | 0.785 |
| *Butyrivibrio* | 0.010 | 0.083 | 0.001 | 0.873 |
| *Anaerostipes* | 0.014 | 0.054 | 0.024 | 0.825 |
| *norank_f__Clostridiales_vadinBB60_group* | 0.005 | 0.058 | 0.021 | 0.709 |
| *Sphaerochaeta* | ND | 0.082 | ND | 0.035 |
| *Lachnospiraceae_ND3007_group* | 0.041 | 0.033 | 0.005 | 0.612 |
| *norank_f__Erysipelotrichaceae* | 0.013 | 0.051 | 0.005 | 0.454 |
| *Ruminococcaceae_UCG-010* | 0.006 | 0.054 | 0.009 | 0.408 |
| *Prevotellaceae_UCG-004* | 0.019 | 0.021 | 0.026 | 0.866 |
| *Ruminiclostridium_6* | 0.017 | 0.036 | 0.005 | 0.709 |
| *Olsenella* | 0.042 | 0.005 | 0.008 | 0.825 |
| *[Eubacterium]_ruminantium_group* | 0.001 | 0.052 | ND | 0.517 |
| *Ruminococcaceae_UCG-009* | 0.004 | 0.047 | ND | 0.405 |
| *Veillonella* | 0.014 | 0.018 | 0.018 | 0.982 |
| *Marvinbryantia* | 0.010 | 0.021 | 0.013 | 0.873 |
| *Paraprevotella* | ND | 0.022 | 0.022 | 0.709 |
| *Lachnospiraceae_FCS020_group* | 0.008 | 0.023 | 0.010 | 0.517 |
| *Erysipelotrichaceae_UCG-003* | ND | 0.037 | ND | 0.398 |
| *Butyricimonas* | 0.002 | 0.035 | ND | 0.709 |
| *Holdemania* | 0.006 | 0.007 | 0.022 | 0.517 |
| *norank_o__Gastranaerophilales* | 0.003 | 0.013 | 0.017 | 0.940 |
| *Anaerofilum* | 0.016 | 0.016 | 0.003 | 0.517 |
| *Intestinimonas* | 0.008 | 0.022 | 0.002 | 0.757 |
| *norank_f__Coriobacteriaceae* | 0.006 | 0.022 | 0.003 | 0.755 |
| *unclassified_f__Erysipelotrichaceae* | 0.006 | 0.018 | 0.003 | 0.866 |
| *Senegalimassilia* | 0.003 | 0.021 | 0.003 | 0.525 |
| *Howardella* | 0.010 | 0.013 | 0.003 | 0.755 |
| *[Eubacterium]_nodatum_group* | 0.003 | 0.021 | 0.001 | 0.709 |
| *Candidatus_Soleaferrea* | 0.001 | 0.021 | 0.002 | 0.494 |
| *Christensenellaceae_R-7_group* | 0.003 | 0.016 | 0.003 | 0.455 |
| *Anaerobiospirillum* | ND | 0.022 | 0.000 | 0.517 |
| *Bifidobacterium* | ND | 0.006 | 0.015 | 0.007 |
| *Anaerovorax* | 0.002 | 0.009 | 0.010 | 0.517 |
| *Mogibacterium* | 0.003 | 0.010 | 0.007 | 0.873 |
| *dgA-11_gut_group* | 0.004 | 0.015 | ND | 0.709 |
| *Allisonella* | 0.007 | 0.007 | 0.005 | 0.866 |
| *norank_f__Peptococcaceae* | 0.003 | 0.015 | ND | 0.709 |
| *Slackia* | 0.006 | 0.004 | 0.007 | 0.866 |
| *Defluviitaleaceae_UCG-011* | 0.003 | 0.007 | 0.003 | 0.785 |
| *[Eubacterium]_ventriosum_group* | ND | 0.008 | 0.004 | 0.709 |
| *Succinivibrio* | 0.010 | 0.001 | ND | 0.848 |
| *norank_c__Cyanobacteria* | 0.004 | 0.004 | 0.002 | 0.785 |
| *Selenomonas* | ND | ND | 0.010 | 0.709 |
| *Kitasatospora* | 0.004 | 0.003 | 0.003 | 0.873 |
| *Catenisphaera* | ND | 0.010 | ND | 0.709 |
| *Fibrobacter* | ND | 0.009 | ND | 0.709 |
| *Anaeroplasma* | ND | 0.007 | ND | 0.709 |
| *Erysipelotrichaceae_UCG-004* | ND | 0.005 | ND | 0.709 |
| *Leptotrichia* | 0.003 | ND | 0.002 | 0.709 |
| *Enterorhabdus* | 0.003 | ND | 0.001 | 0.709 |
| *unclassified_f__Family_XIII* | ND | 0.002 | ND | 0.517 |
| *[Anaerorhabdus]_furcosa_group* | ND | 0.002 | ND | 0.709 |
| *unclassified_o__Clostridiales* | ND | ND | 0.002 | 0.709 |
| *Mucispirillum* | 0.001 | 0.001 | ND | 0.848 |
| *Coprococcus_2* | 0.001 | ND | ND | 0.709 |

“ND”, not detected.
