# Supplementary material for: Lactobacillus plantarum PFM 105 Promotes Intestinal Development Through Modulation of Gut Microbiota in Weaning Piglets
Source: Front Microbiol. 2019 Feb 5;10:90. doi: 10.3389/fmicb.2019.00090 (PMC6371750; doi:10.3389/fmicb.2019.00090)
Supplement: Supplementary file 4 [file Table_4.DOCX]

***Lactobacillus plantarum* PFM 105 promotes intestinal development through modulation of gut microbiota** **in weaning piglets**

**Tianwei Wang^1,2^†, Kunling Teng^1^†, Yayong Liu^1,2^, Weixiong Shi^1,2^, Jie Zhang^1^, Enqiu Dong^3^, Xin Zhang^3^, Yong Tao^1,2^, Jin Zhong^1,2*^**

^1^ State Key Laboratory of Microbial Resources, Institute of Microbiology, Chinese Academy of Sciences, Beijing, China

^2^ University of Chinese Academy of Sciences, Beijing, China

^3^ LongDa Foodstuff Group Co., Ltd, Shandong Province, China

***Correspondence:**

Jin Zhong

[zhongj@im.ac.cn](mailto:zhongj@im.ac.cn)

Table S4. Relative abundance of predicted functions for specific KEGG (Kyoto Encyclopedia of Genes and Genomes) modules (level 1) according to histology.

| **KEGG modules Level 1** | **NC** | **PC** | **LP** | ***P* Value** |
| --- | --- | --- | --- | --- |
| Cellular Processes | 3.16±0.73 | 3.07±0.34 | 2.56±0.53 | 0.163 |
| Environmental Information Processing | 10.56±0.94 | 11.46±0.34 | 9.59±0.92 | 0.003 |
| Genetic Information Processing | 21.78±0.43 | 21.69±0.5 | 22.06±0.45 | 0.38 |
| Human Diseases | 0.77±0.01 | 0.78±0.07 | 0.78±0.02 | 0.828 |
| Metabolism | 48.81±1.15 | 48.03±0.47 | 49.99±1 | 0.008 |
| Organismal Systems | 0.86±0.02 | 0.79±0.05 | 0.85±0.03 | 0.012 |
| Unclassified | 13.86±0.23 | 13.99±0.34 | 13.97±0.15 | 0.623 |
