# Supplementary material for: Lactobacillus plantarum PFM 105 Promotes Intestinal Development Through Modulation of Gut Microbiota in Weaning Piglets
Source: Front Microbiol. 2019 Feb 5;10:90. doi: 10.3389/fmicb.2019.00090 (PMC6371750; doi:10.3389/fmicb.2019.00090)
Supplement: Supplementary file 5 [file Table_5.DOCX]

***Lactobacillus plantarum* PFM 105 promotes intestinal development through modulation of gut microbiota** **in weaning piglets**

**Tianwei Wang^1,2^†, Kunling Teng^1^†, Yayong Liu^1,2^, Weixiong Shi^1,2^, Jie Zhang^1^, Enqiu Dong^3^, Xin Zhang^3^, Yong Tao^1,2^, Jin Zhong^1,2*^**

^1^ State Key Laboratory of Microbial Resources, Institute of Microbiology, Chinese Academy of Sciences, Beijing, China

^2^ University of Chinese Academy of Sciences, Beijing, China

^3^ LongDa Foodstuff Group Co., Ltd, Shandong Province, China

***Correspondence:**

Jin Zhong

[zhongj@im.ac.cn](mailto:zhongj@im.ac.cn)

Table S5. Relative abundance of predicted functions for specific KEGG modules (level 2) according to histology. KEGG, Kyoto Encyclopedia of Genes and Genomes.

| **KEGG modules Level 2** | **NC** | **PC** | **LP** | ***P* Value** |
| --- | --- | --- | --- | --- |
| Amino Acid Metabolism | 9.8±0.24 | 9.76±0.18 | 9.97±0.29 | 0.306 |
| Biosynthesis of Other Secondary Metabolites | 1.05±0.07 | 0.99±0.09 | 1.12±0.07 | 0.036 |
| Carbohydrate Metabolism | 9.82±0.28 | 9.59±0.51 | 9.94±0.35 | 0.322 |
| Energy Metabolism | 6.04±0.21 | 6.04±0.1 | 6.2±0.17 | 0.169 |
| Enzyme Families | 2.36±0.07 | 2.34±0.07 | 2.35±0.06 | 0.795 |
| Glycan Biosynthesis and Metabolism | 2.98±0.31 | 2.83±0.18 | 3.31±0.31 | 0.220 |
| Lipid Metabolism | 2.66±0.04 | 2.63±0.06 | 2.56±0.07 | 0.032 |
| Metabolism of Cofactors and Vitamins | 4.88±0.09 | 4.73±0.05 | 5.02±0.13 | 0.0003 |
| Metabolism of Other Amino Acids | 1.57±0.06 | 1.54±0.02 | 1.64±0.05 | 0.003 |
| Metabolism of Terpenoids and Polyketides | 1.83±0.05 | 1.78±0.03 | 1.88±0.07 | 0.016 |
| Nucleotide Metabolism | 4.58±0.17 | 4.51±0.11 | 4.73±0.19 | 0.090 |
| Xenobiotics Biodegradation and Metabolism | 1.34±0.06 | 1.4±0.06 | 1.36±0.04 | 0.163 |
| Folding, Sorting and Degradation | 2.74±0.08 | 2.7±0.09 | 2.8±0.08 | 0.117 |
| Replication and Repair | 9.98±0.3 | 9.89±0.28 | 10.22±0.32 | 0.182 |
| Transcription | 2.7±0.06 | 2.74±0.09 | 2.58±0.12 | 0.033 |
| Translation | 6.4±0.14 | 6.41±0.23 | 6.5±0.18 | 0.633 |
| Membrane Transport | 9.05±0.81 | 9.93±0.28 | 8.22±0.79 | 0.002 |
| Signal Transduction | 1.34±0.18 | 1.39±0.07 | 1.19±0.17 | 0.081 |
| Signaling Molecules and Interaction | 0.19±0.02 | 0.17±0.02 | 0.2±0.02 | 0.019 |
| Cellular Processes and Signaling | 4.13±0.09 | 4.16±0.12 | 4.1±0.08 | 0.500 |
| Genetic Information Processing | 2.65±0.06 | 2.69±0.12 | 2.66±0.08 | 0.760 |
| Metabolism | 2.35±0.14 | 2.34±0.09 | 2.49±0.17 | 0.150 |
| Poorly Characterized | 4.75±0.1 | 4.82±0.17 | 4.76±0.11 | 0.591 |
| Cell Motility | 2.29±0.82 | 2.24±0.34 | 1.65±0.59 | 0.171 |
| Transport and Catabolism | 0.3±0.07 | 0.27±0.02 | 0.31±0.04 | 0.330 |
