# Supplementary material for: Lactobacillus plantarum PFM 105 Promotes Intestinal Development Through Modulation of Gut Microbiota in Weaning Piglets
Source: Front Microbiol. 2019 Feb 5;10:90. doi: 10.3389/fmicb.2019.00090 (PMC6371750; doi:10.3389/fmicb.2019.00090)
Supplement: Supplementary file 6 [file Table_6.DOCX]

***Lactobacillus plantarum* PFM 105 promotes intestinal development through modulation of gut microbiota** **in weaning piglets**

**Tianwei Wang^1,2^†, Kunling Teng^1^†, Yayong Liu^1,2^, Weixiong Shi^1,2^, Jie Zhang^1^, Enqiu Dong^3^, Xin Zhang^3^, Yong Tao^1,2^, Jin Zhong^1,2*^**

^1^ State Key Laboratory of Microbial Resources, Institute of Microbiology, Chinese Academy of Sciences, Beijing, China

^2^ University of Chinese Academy of Sciences, Beijing, China

^3^ LongDa Foodstuff Group Co., Ltd, Shandong Province, China

***Correspondence:**

Jin Zhong

[zhongj@im.ac.cn](mailto:zhongj@im.ac.cn)

Table S6. Composition of experimental diets and nutritional values (as basis feed).

| **Item** | **Concentration (w/w) %** |
| --- | --- |
| **Ingredients** |  |
| Corn, 8% CP^1^ | 38.8 |
| Soybean meal, 46% CP | 21 |
| Barley, 12% CP | 2 |
| Whey flour, 12% CP | 5 |
| Extruded soybean | 5 |
| Fat power | 2 |
| White granulated sugar | 2.5 |
| Glucose | 2.5 |
| Soybean oil | 2 |
| Fishmeal, 62.5% | 3.5 |
| Dried whey | 10 |
| Dicalcium phosphate | 0.74 |
| Calcium carbonate | 0.5 |
| Salt | 0.3 |
| Limestone | 0.36 |
| Lysine | 0.6 |
| Methionine | 0.25 |
| Threonine | 0.36 |
| Tryptophan | 0.09 |
| Vitamin-mineral premix^2^ | 2.5 |
| Total | 100 |
| **Analyzed chemical composition** |  |
| Dry Matter | 90 |
| CP | 19 |
| P | 0.6 |
| Ca | 0.7 |
| Lysine | 1.5 |
| Methionine+ Cysteine | 0.85 |
| Threonine | 1 |
| Tryptophan | 0.3 |
| Net energy (MJ/kg) | 10.5 |

^1^CP: Crude proteins.

^2^Vitamin-mineral premix: Premix provided the following per kg: vitamin A,19,200 IU; vitamin D3, 4,800 IU; vitamin E, 60 IU; vitamin K3, 6 mg; vitamin B1, 6 mg; vitamin B2, 12 mg; vitamin B6, 7.2 mg; vitamin B12, 0.05 mg; niacin, 60 mg; calcium pantothenate, 30 mg; nicotinic acid, 15 mg; folic acid, 3.60 mg; biotin, 0.60 mg; Fe, 305 mg; Cu, 250 mg; Zn, 1,910 mg; Mn, 51 mg; I, 0.50 mg; Se, 0.50 mg; Co, 0.50 mg.
