# Supplementary material for: Lactobacillus plantarum PFM 105 Promotes Intestinal Development Through Modulation of Gut Microbiota in Weaning Piglets
Source: Front Microbiol. 2019 Feb 5;10:90. doi: 10.3389/fmicb.2019.00090 (PMC6371750; doi:10.3389/fmicb.2019.00090)
Supplement: Supplementary file 7 [file Table_7.DOCX]

***Lactobacillus plantarum* PFM 105 promotes intestinal development through modulation of gut microbiota** **in weaning piglets**

**Tianwei Wang^1,2^†, Kunling Teng^1^†, Yayong Liu^1,2^, Weixiong Shi^1,2^, Jie Zhang^1^, Enqiu Dong^3^, Xin Zhang^3^, Yong Tao^1,2^, Jin Zhong^1,2*^**

^1^ State Key Laboratory of Microbial Resources, Institute of Microbiology, Chinese Academy of Sciences, Beijing, China

^2^ University of Chinese Academy of Sciences, Beijing, China

^3^ LongDa Foodstuff Group Co., Ltd, Shandong Province, China

***Correspondence:**

Jin Zhong

[zhongj@im.ac.cn](mailto:zhongj@im.ac.cn)

Tables S7. Sequences of oligonucleotides used in this study and lengths of the respective PCR products.

| **Bacterial genes** | **Primer** | | **Product size (bp)** | **Reference** |
| --- | --- | --- | --- | --- |
|  | Direction | Sequences (5‘→3’) |  |  |
| 16S rRNA gene | 27F | AGAGTTTGATCCTGGCTCAG | 1466 bp | (Gao, Ma et al. 2017) |
|  | 1492R | GGTTACCTTGTTACGACTT |  |  |
| V3-V4 region of 16S rRNA gene | 338F | ACTCCTACGGGAGGCAGCA | 468 bp | (Cheng, Wei et al. 2018) |
|  | 806R | GGACTACHVGGGTWTCTAAT |  |  |
