# Supplementary material for: Lactobacillus plantarum PFM 105 Promotes Intestinal Development Through Modulation of Gut Microbiota in Weaning Piglets
Source: Front Microbiol. 2019 Feb 5;10:90. doi: 10.3389/fmicb.2019.00090 (PMC6371750; doi:10.3389/fmicb.2019.00090)

***Lactobacillus plantarum* PFM 105 promotes intestinal development through modulation of gut microbiota** **in weaning piglets**

**Tianwei Wang^1,2^†, Kunling Teng^1^†, Yayong Liu^1,2^, Weixiong Shi^1,2^, Jie Zhang^1^, Enqiu Dong^3^, Xin Zhang^3^, Yong Tao^1,2^, Jin Zhong^1,2*^**

^1^ State Key Laboratory of Microbial Resources, Institute of Microbiology, Chinese Academy of Sciences, Beijing, China

^2^ University of Chinese Academy of Sciences, Beijing, China

^3^ LongDa Foodstuff Group Co., Ltd, Shandong Province, China

***Correspondence:**

Jin Zhong

[zhongj@im.ac.cn](mailto:zhongj@im.ac.cn)

Figure S1. Alpha diversity analysis of the colonic microbiota. Rarefaction curves for OTUs (A) and Shannon index (B), as well as rank-abundance curves (C) of each sample were calculated for reads exhibiting ≥97% sequence identity.

**
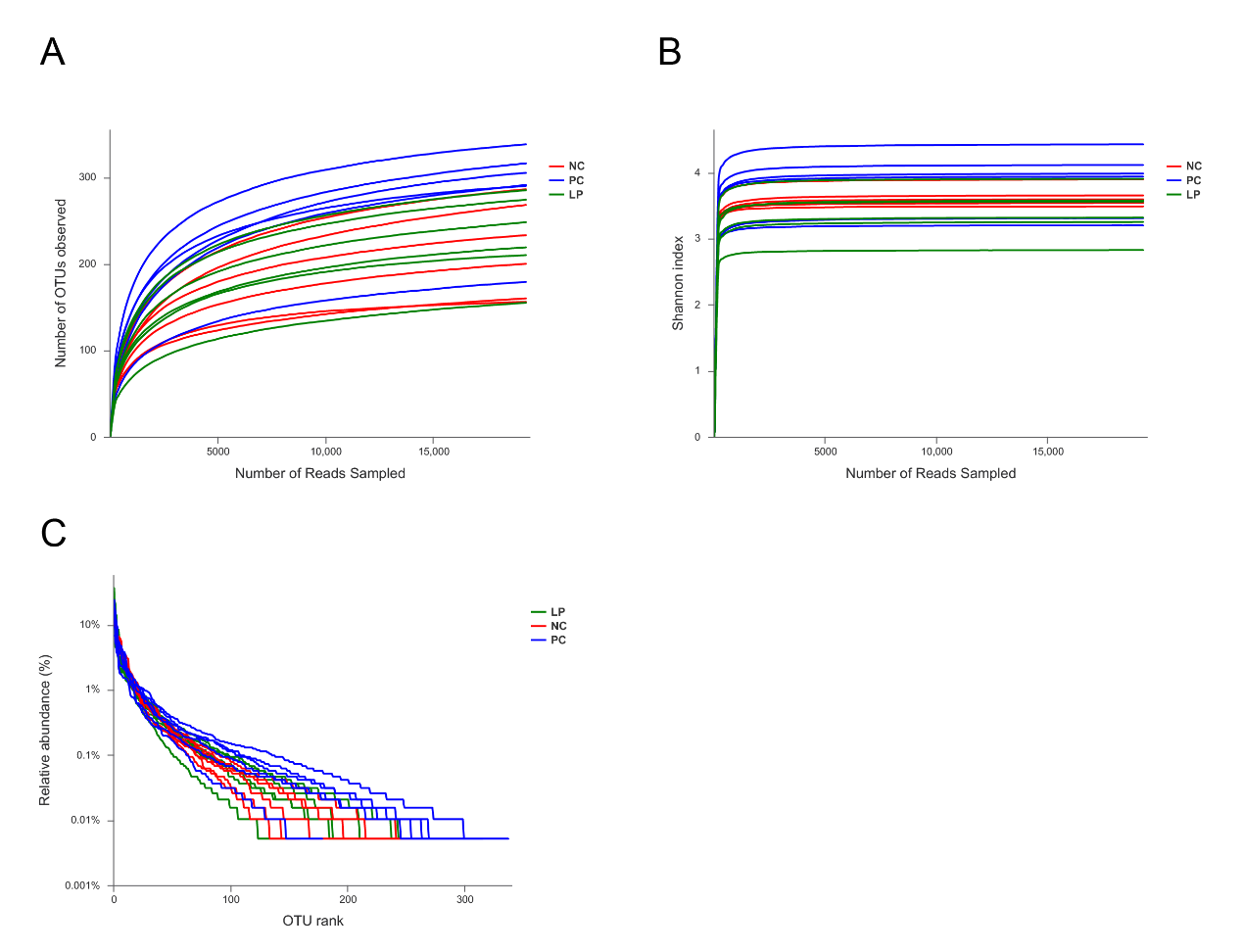
**

Figure S2. Venn diagram of OTUs of weaning piglets treated with antibiotics (PC) and *L. plantarum* (LP).

**
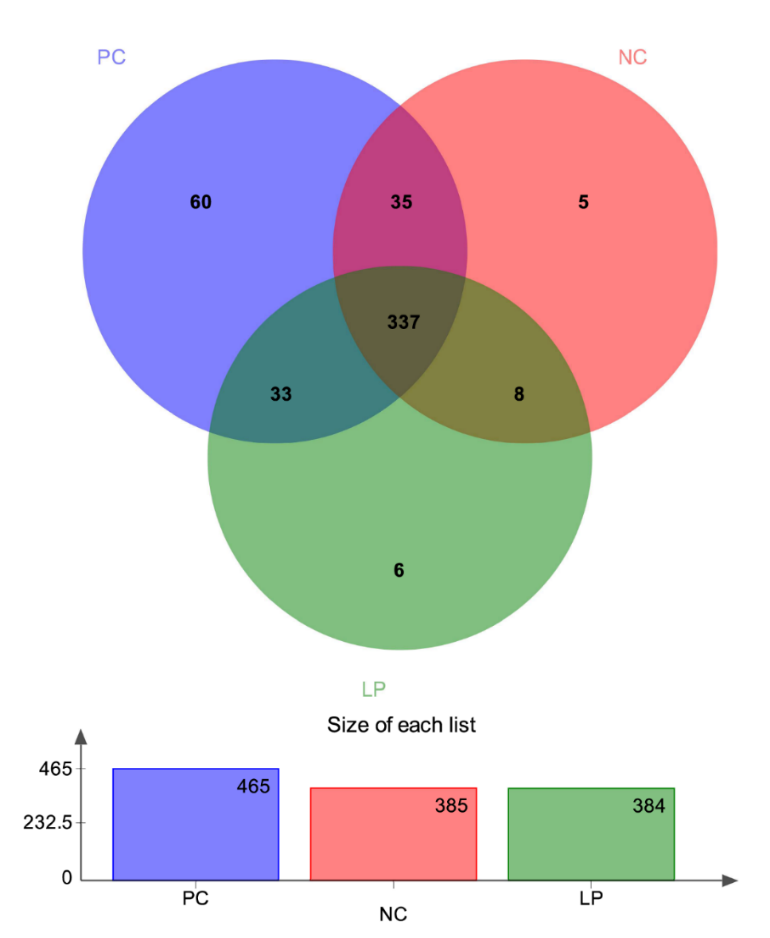
**

Figure S3. Beta diversity analysis of the colonic microbiota. (A) Hierarchical clustering tree of the microbial community based on Unweighted Unifrac distances. (B) Principal component analysis at the phylum level. (C) Principal component analysis at the OTU level.


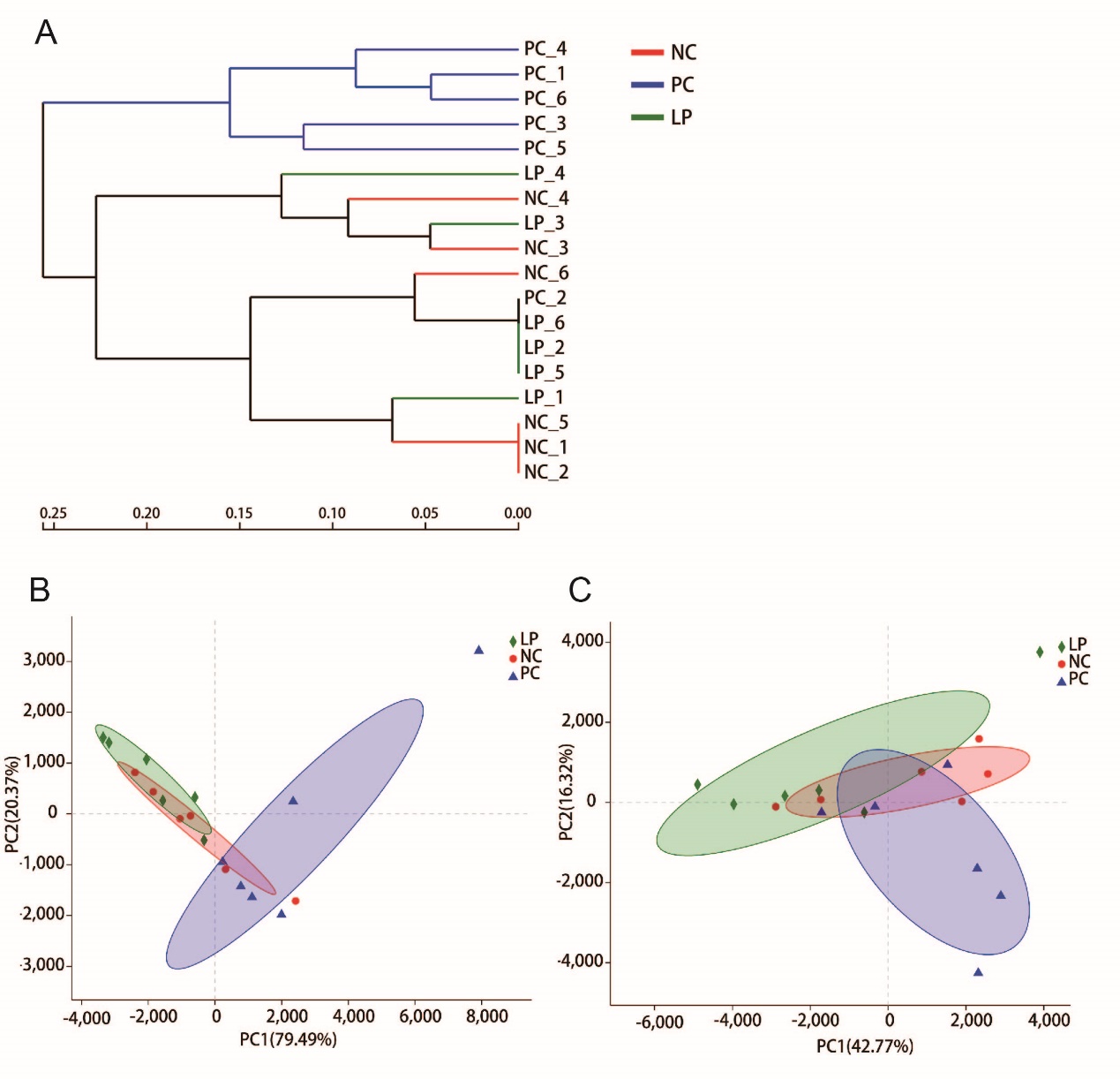

Supplement: Supplementary file 8 [file Table_8.DOCX]
